# Discovery of two novel small-molecule series with potent SARS-CoV-2 inhibition *via* putative nsp13 open-state trapping

**DOI:** 10.1039/d6sc02048h

**Published:** 2026-08-28

**Authors:** Alma C. Castañeda-Leautaud, Thomas D. Bannister, Eunjung Kim, Donghoon Chung, Rommie E. Amaro

**Affiliations:** a Department of Chemistry and Biochemistry, University of California San Diego La Jolla CA 92093-0533 USA ramaro@ucsd.edu +1 (858) 534-9629; b Department of Molecular Medicine, The Herbert Wertheim UF Scripps Institute for Biomedical Innovation and Technology Jupiter FL 33458 USA; c Department of Microbiology and Immunology, University of Louisville Louisville KY 40292 USA

## Abstract

We report the discovery of 31 SARS-CoV-2 inhibitors identified across two computational-to-experimental screening campaigns, with average cell-based antiviral potency satisfying 〈EC_50_〉 ≤ CC_50_/3 in A549-hACE2 cells. These compounds were selected from 60 successfully tested molecules evaluated using nanoluciferase reporter assay (nLuc), cytopathic effect (CPE), and host-cell cytotoxicity (CC_50_) assays. We used a funnel-like computational framework where candidate compounds were progressively prioritized through different scores, improving robustness against the limitations of individual metrics. However, none of the docking-based scoring metrics, including MM/GBSA and Glide scores, showed statistically significant correlation with experimental activity, illustrating the limitations of docking-only approaches and supporting a major contribution of the machine-learning predictions to the high hit-identification rate. Although direct target validation is still lacking, the machine-learning (ML) workflow was grounded in experimental nsp13 inhibition labels, and molecular dynamics (MD) simulations suggest that these compounds can act by stabilizing apo-like open conformations of nsp13, with differential interactions involving the 1B domain contributing to potency differences. Among the compounds evaluated, the phenoxypropanol (PP) and bipiperidine (BPP) series stood out as the most promising SARS-CoV-2 antivirals. Integrated analysis of structure–activity relationships, physicochemical parameters, metabolic stability, and interdomain dynamics delineated structural optimization paths for both series.

## Abstract 2

We report the discovery of 31 SARS-CoV-2 inhibitors identified across two computational-to-experimental screening campaigns, with average cell-based antiviral potency satisfying 〈EC_50_〉 ≤ CC_50_/3 in A549-hACE2 cells.

## Graphical abstract

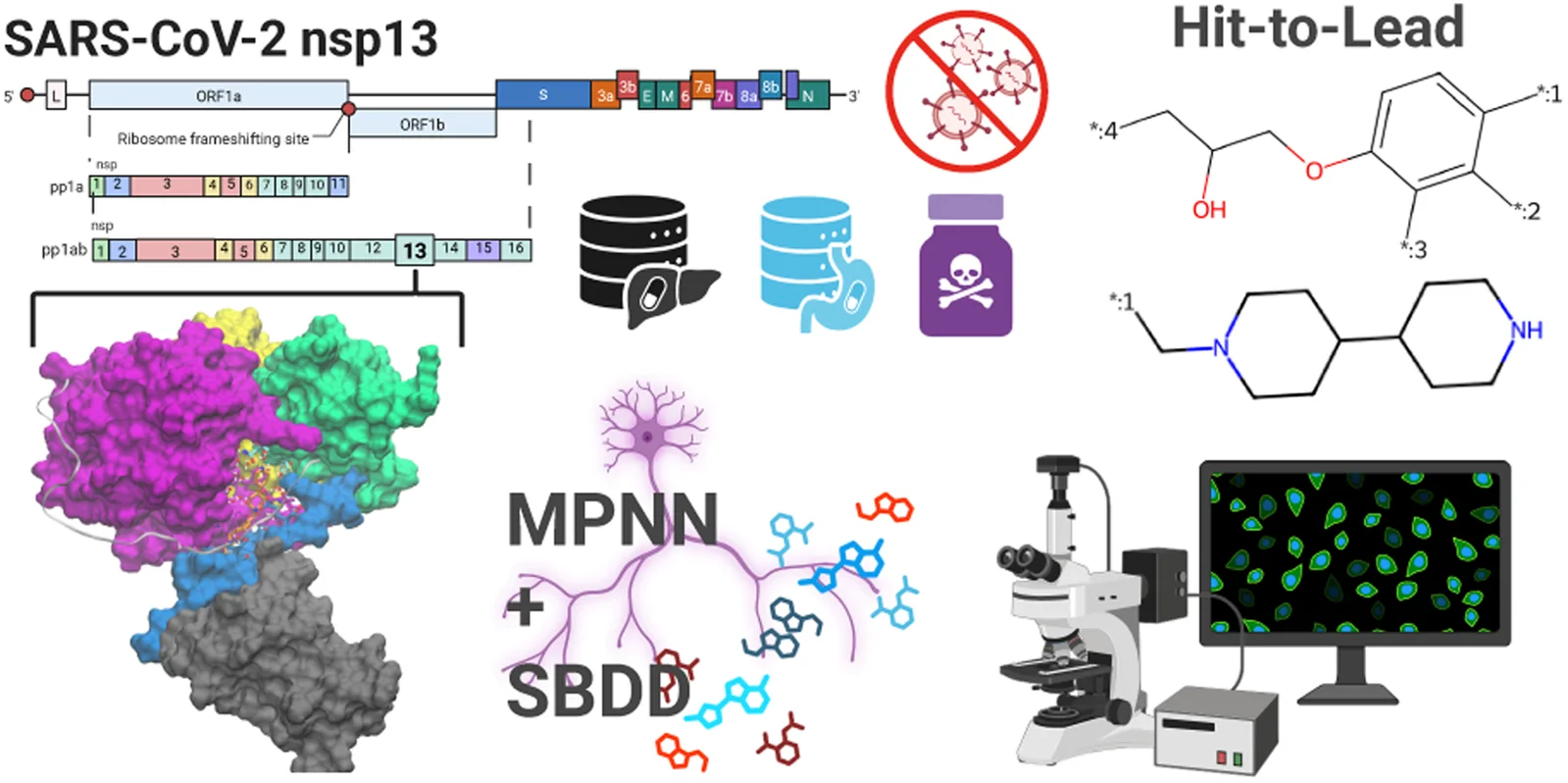

## 1 Introduction

Severe acute respiratory syndrome coronavirus 2 (SARS-CoV-2), the causative agent of coronavirus disease 2019 (COVID-19), has caused nearly 7 million deaths worldwide since its emergence in December 2019.^1^

Nonstructural proteins (nsps) are attractive antiviral targets because they are essential for replication. Most antiviral strategies have focused on inhibiting the polymerase and protease nsps.^2^ Recently, nsp13, a viral helicase, has emerged as a promising target due to its high conservation across beta- and alpha-coronaviruses,^3^ offering broad-spectrum activity. This ATP-dependent enzyme is crucial for viral replication and transcription, unwinding, translocating, and backtracking double- and single-stranded DNA and RNA in the 5′ to 3′ direction.^4,5^

Importantly, a multiple-sequence alignment of Omicron XBB.1 and the currently circulating COVID-19 Variants Under Monitoring (VUMs) and Variants of Interest (VOIs) indicates that nsp13 is approximately 99.6% conserved relative to the original Wuhan-HU-1 strain (WHO, 2025). None of the observed mutations occur within the RNA-binding channel of nsp13, making this region the most conserved binding site among the COVID-19 nsps.^6^

Structurally, nsp13 adopts a triangular shape, comprising two RecA-like (Rec1A and Rec2A) and 1B domains, whose interface forms the RNA-binding channel.^5^ Additional domains include the stalk and a zinc-binding domain (ZBD) containing three native zinc ions: ZF1 (composed by Cys5, Cys8, Cys26, and Cys29), ZF2 (Cys16, Cys19, His33, and His39), and ZF3 (Cys50, Cys55, Cys72, and His75).^7^ To date, only a limited number of nsp13 inhibitors have been reported,^8,9^ despite the identification of several putative binding pockets through crystallographic fragment screening.^6^ This limited success reflects the substantial conformational flexibility of the helicase interdomain architecture, particularly motions involving the 1B domain during RNA translocation.

In response to the lack of effective antiviral drugs, the Midwest Antiviral AViDD Center, tasked with exploring approaches to target pathogens with pandemic potential, including SARS-CoV-2 nsp13. The project started with a high-throughput screening (HTS) campaign that tested approximately 650 000 compounds from a highly diverse dataset using a FRET-based fluorescence assay. This assay measured the unwinding activity of nsp13 by detecting changes in fluorescence from a FAM-labeled DNA substrate in the presence of trap DNA and ATP.^10^ Among the various hits identified, we discovered the THIQ inhibitor series.^11^

**Fig. 1:**
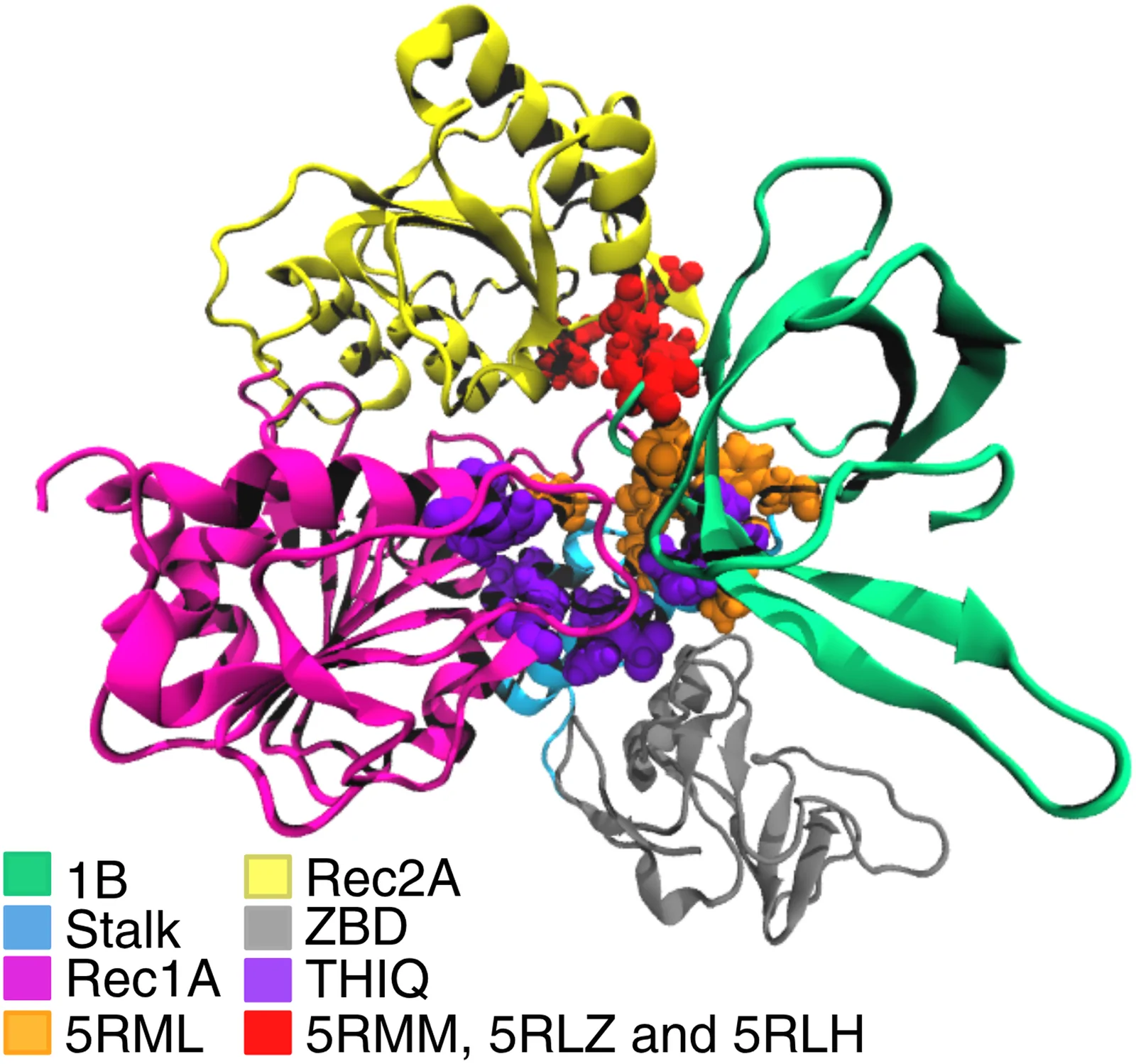
The nsp13 helicase comprises the zinc-binding (ZBD; residues 1–100), stalk (101–150), 1B (151–226), Rec1A (260–441), and Rec2A (444–601) domains, shown in distinct colors. van der Waals (vdW) surface representations highlight residues defining reported fragment and ligand binding sites. The PDB 5RML site (orange) involves Stalk residues E142, F145, K146, and Y149; 1B residue Y180; and Rec1A residues L227, P408, and T410. The fragment-binding pocket shared by PDBs 5RMM, 5RLZ, and 5RLH (red) is located at the Rec2A–1B interface and includes N177, H482, S485, S486, N516, T552, and H554.^6^ The tetrahydroisoquinoline (THIQ) series binding site (purple), comprising 18 compounds, overlaps substantially with the 5RML site and additionally includes Stalk residue L139; 1B residues A179 and V181; Rec1A residues E228, H230, A338, N361, and T380.^14^

In this work, we target the THIQ binding region extending from the RNA-pocket region,^12^ including the 5RLK fragment site, towards the more buried 5RML fragment site located near the stalk domain.^6^ Notably, the 5RML fragment and our THIQ binders occupy a partially distinct region relative to the clustered RNA-channel fragments (5RMM, 5RLZ, and 5RLH), the principal binding region targeted by most CACHE challenge 2 hits,^13^ highlighting the relative novelty of our stalk-proximal site (Fig. 1).

## 2 Results and discussion

To enable the identification of nsp13 inhibitors from both in-stock Enamine and Chembridge libraries, we first constructed an Undirected Message-Passing Neural Network (UMPNN) and explicitly incorporated three levels of molecular representation: atom-, bond-, and global-level features based on our previous findings.^14^ Only features with low inter-correlation within their respective categories were retained (<0.85; SI, Fig. 1.2) to ensure complementary information across representations. Hyperparameters were set empirically based on reduced overfitting using cross-validation runs (SI, Table 1.2). More details regarding the UMPNN implementation are described in the SI, Section 1.1.

The training data combines 2127 curated HTS data^10^ and 492 cell-based EC_50_ readouts totaling 2619 compounds. This creates a hybrid objective, enabling the model to learn chemotypes beyond isolated enzyme inhibition that also meet cellular constraints, such as cell permeability and physiological solubility.

Cell-based values were binarized using an EC_50_ threshold of 15 µM, yielding 209 active compounds. Binary HTS labels were assigned using the inhibition threshold (22.29% ± SE) defined in the original HTS campaign.^10^ Compounds with ambiguous near-boundary activities were excluded, yielding 519 additional actives.

The final dataset exhibits a moderate class imbalance of 1 : 2.6 and substantial chemical diversity, with an average pairwise Tanimoto similarity of 0.12. In addition, it presents a significant classification challenge, as reflected by a mean maximum nearest-neighbor similarity of 0.50 between class members. The distributions of both similarity metrics are provided in the SI (Fig. 1.3). Two compound pairs displayed a Tanimoto similarity of 1.0 despite not being duplicate entries.

### 2.1 Structure-based funnel-like screening

The scaffold-based (80 : 20 train to test) blind-test of the UMPNN model demonstrated strong discriminative performance, achieving an AUROC of approximately 0.91, indicating effective ranking of active *versus* inactive compounds. In contrast, the more moderate F1 score of around 0.69 suggests that binary classification at the selected decision threshold remained suboptimal, with a considerable number of both false positives and false negatives.

We next analyzed performance across different nearest-neighbor (NN) similarity regimes to assess model dependence on the training data. For each test-set compound, the maximum Tanimoto similarity to any training compound was calculated and used for performance stratification (Table 1).

**Table 1:** Model performance stratified by train–test similarity tiers. Reported values correspond to the mean ± standard error across three repeated runs. The fraction column indicates the proportion of the scaffold-test set contained within each similarity tier. The D/A ratio corresponds to the decoy-to-active ratio. NN ≥0.50 indicates the fraction of compounds within each tier whose nearest opposite-class neighbor had Tanimoto similarity at least 0.50

Train/test sim. tiers	Test fraction	D/A ratio	Active-decoy NN ≥0.50	F1	AUROC	Accuracy	Training time
<0.25	1.5%	3.0	0.0%	33.3% ± 33.3	0.861 ± 0.100	79.2% ± 11.0	—
0.25–<0.50	61.3%	4.8	8.1%	51.1% ± 2.5	0.906 ± 0.007	87.1% ± 0.4	—
0.50–<0.75	33.8%	0.8	51.4%	76.2% ± 3.4	0.812 ± 0.002	74.6% ± 2.3	—
0.75–1.00	3.4%	0.1	0.0%	82.5% ± 9.7	1.000 ± 0.000	74.1% ± 13.0	—
Overall	100.0%	2.6	—	69.7% ± 3.9	0.912 ± 0.003	82.3% ± 1.4	8.92 ± 0.67 min

The model maintained strong ranking performance in the dominant low-to-moderate similarity regime, achieving an AUROC of 0.906 ± 0.007 for compounds with NN similarities between 0.25 and 0.50, although F1 scores were lower, possibly due to class imbalance. Only 3.4% of compounds in the scaffold-test set exhibited NN similarities above 0.75, indicating that the splitting strategy successfully established chemotype independence between the training and test sets.

Nevertheless, the majority of active compounds clustered within the 0.50–0.75 similarity range, reflecting the limited diversity of hit scaffolds. This interval showed the lowest AUROC values, due to greater structural overlap between active and decoy compounds, with 51.4% of molecules having an opposite-class nearest neighbor at similarity ≥0.50, highlighting the increased classification difficulty in this chemically ambiguous region.

The final prediction set was composed of 1 172 240 commercial drug-like compounds. The model predicted 1866 actives, corresponding to roughly 0.16% of the initial set. To assess the novelty reach of the UMPNN predictions, we quantified chemical similarity between the predicted inhibitors and the training set. The NN similarity distribution showed a median similarity of 0.28, with only 7.6% and 1.5% of predictions exceeding similarity thresholds of 0.4 and 0.5, respectively. These results demonstrate that the majority of predicted compounds lie outside close analogue space.

The next step in the funnel-like strategy involved molecular docking. The predicted inhibitors were expanded to all possible stereoisomers and protonation states. This preparation expanded our candidates to 9947 conformations, which were docked to the THIQ site^11^ of the nsp13 using Glide.^15^

The lowest EMODEL scoring pose per ligand was retained to select the most optimal conformation for binding. In parallel, compounds with Glide docking scores < −7.5 were selected, resulting in approximately 1064 candidates. MM/GBSA calculations were subsequently performed to re-score the retained binding poses. A total of 353 compounds exhibited MM/GBSA binding free energies more favorable than that of our previously identified nsp13 inhibitor MWAC-3429 (Δ*G* = −43.7 kcal mol; nLuc EC_50_ = 5.4 µM).^11^ Notably, MM/GBSA binding free energies showed only a weak correlation with Glide docking scores (Pearson *r* = 0.147, *p* = 0.006), suggesting that solvation effects play a significant role at this binding site, as solvation energy represents the principal difference between the two scoring methodologies.

To obtain an experimentally tractable yet structurally representative subset from the 353 predicted candidates, compounds were first clustered using DBSCAN and Tanimoto similarity to maximize chemical diversity. The resulting set was then filtered by Maximum Common Substructure (MCS) similarity to the reference inhibitors MWAC-3429, MWAC-3439, and MWAC-3431.^11^ Based on this procedure, 50 compounds with MCS similarity >0.3 to at least one reference ligand were selected for experimental evaluation, with 43 successfully evaluated across nLuc viral replication, CPE, and cytotoxicity assays.

In this first round, MWAC-3805 and MWAC-3795 emerged as the most potent compounds, exhibiting nanomolar cell-based activity (EC_50_ = 500 nM). Overall, 5 candidates were considered hits based on the selectivity criterion given by,
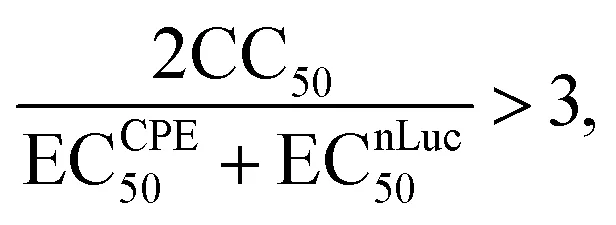


Subsequently, 30 structural analogs of the initial hits, sourced from the ENAMINE and ChemBridge ready-to-purchase libraries, were tested in the same assays. Rather than relying on molecular docking, we prioritized structural similarity metrics to directly expand -R-group exploration around the identified hit scaffolds. In this round, additional 26 hits were identified, where MWAC-3963 was the most potent and least cytotoxic one (CC_50_ > 50 µM, CPE EC_50_ = 766 nM, nLuc EC_50_ = 500 nM).

Overall, 31 of 60 evaluated compounds met the hit criteria (52% hit-identification-rate), indicating that the observed antiviral activity is unlikely to be driven by host-cell toxicity. Hit-identification rate definitions depend on selection criteria, varying widely across studies. Notably, most reports define success solely by EC_50_ or *K*_*i*_ thresholds and overlook cytotoxicity, risking false positives. Here, we report a more stringent biologically validated hit-identification rate of 52%, which remains among the highest for prospective and AI-enhanced VS studies, where typical hit-identification rates range from 1 to 44%.^16–19^

To identify which components of the funnel-like prioritization strategy contributed most strongly to experimental outcomes, we analyzed correlations between computational metrics and biological readouts. Among the evaluated scores, MM/GBSA binding free energies showed the strongest association with CPE EC_50_ values (Pearson *n* = 45, CPE EC_50_*r* = 0.218, *p* = 0.256 and nLuc EC_50_: *r* = 0.061, *p* = 0.693), suggesting only a weak relationship between calculated binding energetics and phenotypic antiviral activity. Glide docking scores exhibited no statistically significant correlation with any experimental measurement, including nLuc (*e.g.*, Glide score *vs.* nLuc EC_50_: *r* = 0.051, *p* = 0.739). These observations suggest that many discarded compounds (up to 1513) represent false negatives. We attribute the poor performance of the docking-based metrics, in part, to the difficulty of adequately sampling the highly solvated and conformationally flexible helicase RNA-binding channel.^5,6^ Collectively, these results suggest that the UMPNN component of the workflow was the primary contributor to the high hit-identification rate.

It is possible that the inclusion of a small portion of nLuc-derived data, corresponding to the same assay later used for hit validation, enabled the model to learn structure–activity relationships directly associated with cell-based replication inhibition. The availability of three previously validated moderately potent hits aided to identify MWAC-3795 retaining the 1-phenoxypropan-2-ol core (PP) scaffold of the previously reported hit MWAC-3439,^11^ however several other validated hits exhibited low structural similarity to compounds in the training set. For example, the closest analogue of MWAC-3805 in the training set is MWAC-3429, yet the two compounds share only low global similarity (Tanimoto = 0.116) and limited structural overlap (36.4% MCS), restricted to a common thiazoline ring. In contrast to the previously identified chemotypes, MWAC-3805 contains a novel 4, 4′-bipiperidine (BPP) scaffold, suggesting that the model captured transferable structure–activity relationships rather than relying solely on close analogue interpolation. Despite this substantial structural divergence, MWAC-3805 retained strong antiviral activity, emphasizing the capacity of the model to extrapolate beyond previously observed chemotypes and preserve mechanistic relevance.

**Fig. 2:**
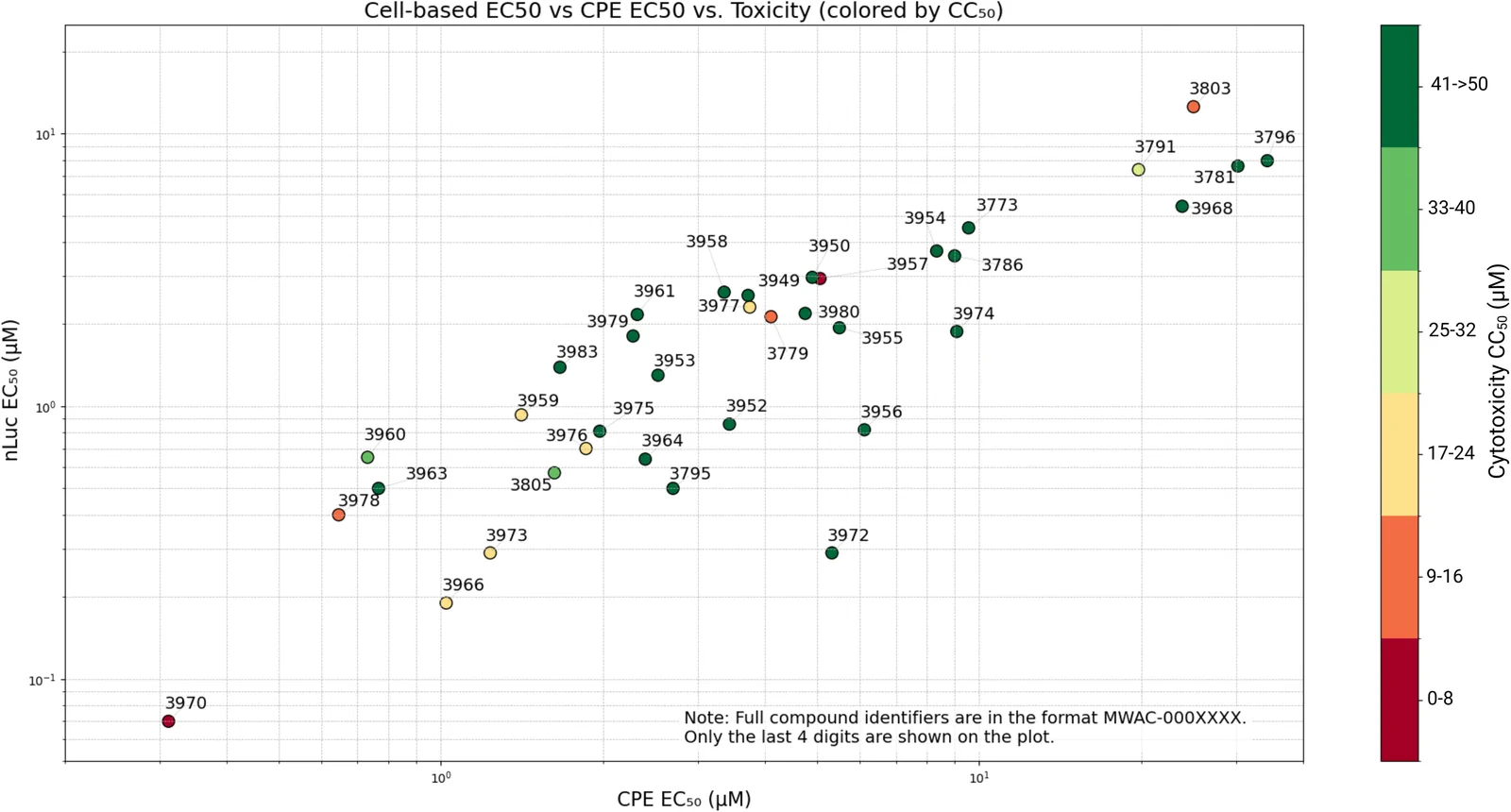
Potency comparison of the two nanomolar series discovered: MWAC-3805, led by MWAC-3972 in terms of nLuc EC_50_ only, and MWAC-3795, led by MWAC-3963 in both assays. Points are color-coded by CC_50_ values (µM), grouped into six tiers.

A representation of the BPP and PP families in terms of orthogonal EC_50_s and CC_50_s space can be observed in Fig. 2. This plot reveals a trend between the two EC_50_ readouts and increased cytotoxic risk among several of the most potent analogs. In particular, MWAC-3970, MWAC-3966, MWAC-3978, and MWAC-3973 exhibited strong antiviral activity but were deprioritized due to unfavorable cytotoxicity profiles. Based on the combined assessment of CPE, nLuc, and CC50 data, MWAC-3960 and MWAC-3963, both analogs of MWAC-3795, emerged as the most balanced candidates. In contrast, MWAC-3972 showed enhanced nLuc potency relative to its reference compound MWAC-3805, but at the expense of reduced CPE activity, illustrating a trade-off between replication inhibition and phenotypic antiviral efficacy in this case.

### 2.2 Binding modes of the two series hits

In the absence of experimental structural or biophysical evidence for direct nsp13 engagement, we characterized the binding modes of two lead chemotypes, MWAC-3972 (BPP) and MWAC-3963 (PP), under the working hypothesis that they target nsp13.

To position these chemotypes within the functional landscape of nsp13, we first assessed whether their predicted binding poses were compatible with conformational rearrangements associated with the ATP hydrolysis cycle. This was accomplished *via* multi-state and multi-site docking, leveraging all relevant crystallographic structures available. After putative binding sites were identified, we conducted molecular simulations of the inhibitor–protein complexes and performed detailed non-bonded interaction analyses to inform and guide subsequent structure–activity relationship (SAR) optimization.

#### 2.2.1 Multi-state docking of the two lead series across the NTP hydrolysis cycle

The NTP hydrolysis cycle involves multiple transitional states that induce major structural rearrangements at the RNA-binding interface, potentially occluding or exposing putative active sites. For this reason, both hits were docked to four distinct nsp13 conformational states across the NTP hydrolysis cycle: (i) an apo state derived from molecular dynamics using PDB ID: 5ROB as the starting reference; (ii) an ATP-like bound state based on PDB ID: 7NN0 (AMP-PNP–bound); (iii) an ADP + P–bound state derived from the nsp13 component of the replication–transcription complex (RTC; PDB ID: 7KRO); and (iv) an nsp13_2_ RNA-bound state from the mini-RTC system (PDB ID: 7CXM).

For each state, the three top-ranked binding sites were selected based on Dscores (SiteMap), and subsequent docking of MWAC-3963 and MWAC-3972 was performed using Glide (SP).^15^

**Fig. 3:**
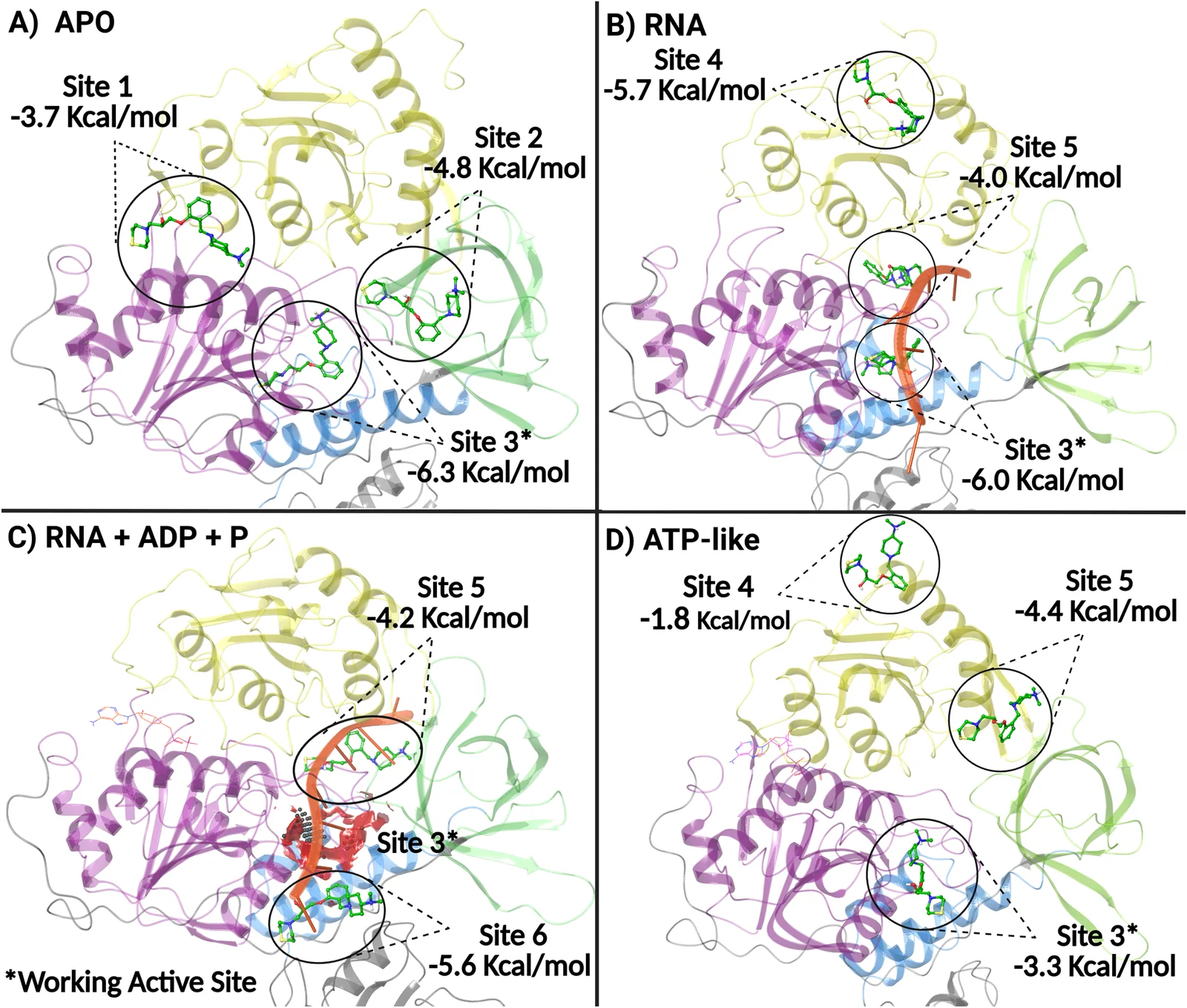
Docking scores for the top three Dscore sites for each of the four conformational states considered. The sites are numbered across the states, with the same number corresponding to the same site between two states. Docking scores of MWAC-3963 are shown per site (A) three sites with Site 1* showing the highest docking score in the Apo state average structure from molecular dynamics simulations. (B) RNA-bound state modeled from PDB ID 7CXM. (C) RNA–ADP–phosphate state based on PDB ID 7KRO. (D) AMP–PNP (ATP analog) bound state from PDB ID 7NN0. Glide docking scores are shown in kcal mol^−1^ for each site.

The results of the multi-docking study are presented in Table 2 and the docking sites can be visualized in Fig. 3 for the docked MWAC-3963.

**Table 2:** Multi-state SiteMap and Glide docking scores (Gscores) for MWAC-3972 and MWAC-3963 across nsp13 conformational states. Sites marked with an asterisk were used in the virtual screening protocol. Dashed entries in the Gscore columns indicate failed docking at the corresponding site, congruent with constriction of the RNA-binding channel across the hydrolysis cycle

ID	State	Dscore	Volume (Å^3^)	Gscore 3972	Gscore 3963
Site-1*	Apo	1.067	367.0	−6.9	−6.3
Site-1*	RNA	1.109	513.9	−6.1	−6.0
Site-2	Apo	1.050	643.5	−5.1	−4.8
Site-3	RNA	1.095	454.7	−5.1	−4.0
Site-1	ADP + RNA	1.062	411.8	−4.8	−5.6
Site-2	ATP-like	1.092	350.9	−4.0	−4.4
Site-2	ADP + RNA	1.052	719.7	−3.8	−4.2
Site-1*	ATP-like	1.117	786.5	−3.8	−3.3
Site-3	Apo	0.981	445.9	−3.4	−3.7
Site-3	ATP-like	0.720	95.0	−2.2	−1.8
Site-2	RNA	1.169	148.3	—	−5.7
Site-3*	ADP + RNA	0.873	129.3	—	—

Both MWAC-3972 and MWAC-3963 achieve their most favorable docking scores in the apo and RNA-bound states (Gscores −6 to −7 kcal mol^−1^) on our working active site. The failure of docking in the ADP + RNA state Site-1 is attributable to pronounced volume collapse: the corresponding pocket measures 513.9 Å^3^ in the RNA-only structure but shrinks to just 129.3 Å^3^ upon ADP binding. Consequently, the closest ligand pose was identified in a neighboring pocket (Site 1, Fig. 3C) located near the 3′ end of the RNA. This pose engages with the Rec1A loop (ALA407–THR410) from the original active site and yielded a docking score of −5.56 kcal mol^−1^, a score substantially lower than the one obtained for the apo state.

The low docking scores observed for the ATP-bound conformation at the active site suggest that these hits are unlikely to bind stably, indicating a transient or weakly formed binding pocket. Collectively, these findings suggest that ligand binding is favored in the open, pre-hydrolytic conformations of nsp13, where the pocket remains accessible, but becomes sterically occluded upon transition to the closed state. Although this site encompasses residues of the RNA binding site previously explored in broader community efforts as RNA competitors, the ability to generate stable poses in the presence of RNA suggests that these compounds act as interfacial or allosteric inhibitors, rather than direct RNA competitors, potentially stabilizing conformations incompatible with productive translocation.^20^ Moreover, the measured solvent-accessible surface area (SASA) of the residues forming the putative active site (residues 120, 139–146, 178–181, 230, 309–312, 361, and 379–384) totals 14.25 nm^2^ in the protein-only model (PDB ID: 7CXM, chain E). Upon RNA binding, approximately 41% of this loop surface area becomes buried at the interface (RNA-bound SASA: 8.36 nm^2^ for PDB ID: 7CXM, chains E and L). Despite this reduction, the pocket remains sufficiently accessible in both conformational states to accommodate the two hit compounds (MW < 394 g mol^−1^), whose physicochemical properties, including low TPSA (<40 Å^2^), moderate lipophilicity (log *P* ≈ 2–3), and limited molecular flexibility, are fully compatible with entering and stabilizing within a partially buried cavity of this size.

Because the ligands were predicted to bind within an interfacial pocket at the helicase–RNA junction, we next assessed whether this interaction resulted in inhibition of RNA unwinding *in vitro*. Using a double-stranded RNA substrate, compounds MWAC-3775, -3778, −3796, −3798, and −3974 exhibited EC_50_ values below 50 µM in the nsp13 unwinding assay. Among these, only MWAC-3974 (BPP series) and MWAC-3796 (PP series) demonstrated measurable antiviral activity in the cell-based nLuc assay. These findings therefore provide only partial support for direct inhibition of nsp13 duplex unwinding and stress a discrepancy between the biochemical and cellular measurements.

Because the UMPNN model was trained on labels specific to nsp13 activity, the same experimental framework that currently classifies our binders as inactive, and because our predicted candidates consistently show favorable docking scores at the proposed binding site, we hypothesize that these compounds may still act as nsp13 binders.

The discrepancy between the biochemical unwinding assay and the cell-based antiviral assays may have several causes. A likely explanation is the lower sensitivity or dynamic range of the biochemical assay compared with the cellular nLuc reporter system. In the nLuc assay, compounds were incubated with infected cells for 48 hours, allowing inhibitory effects to accumulate over multiple viral replication cycles. In contrast, the unwinding assay measured inhibition over <1 hour, during which readouts fluctuated greatly and sometimes exceeded those of the high positive control (Appendix A1), indicating assay instability and potential artifacts. Another possibility is that the unwinding assay measures the helicase in isolation and therefore does not capture its regulatory behavior within the RTC.^5^ Under this scenario, compounds perturbing nsp13 function specifically within the RTC could remain undetected in the isolated assay while still impair viral replication in cells.

Alternatively, compounds displaying measurable unwinding inhibition may suffer from limited cellular permeability or intracellular exposure, preventing validation in the nLuc assay. This may be the case for MWAC-3775, given its strong unwinding activity (EC_50_ = 19 µM) but lack of measurable antiviral effect in cells. The relatively planar and lipophilic character of this scaffold (Fsp^3^ = 0.3, log *P* = 2.5) may contribute to suboptimal developability properties or reduced intracellular exposure. Consequently, compounds with similar behavior were deprioritized at this stage of the project in favor of candidates with stronger preclinical potential. Nevertheless, MWAC-3775 remains mechanistically interesting due to its comparatively potent unwinding inhibition, distinct scaffold, and lack of detectable cytotoxicity, warranting further investigation.

Given the limitations discussed in our enzymatic assay, definitive nsp13 specificity will require orthogonal approaches such as SPR, ITC, CETSA, mutagenesis, or structural crystallography studies, such as cryo-EM.

Pursuing the nsp13 binding hypothesis and based on previous docking studies, we aimed to characterize the binding modes and conformational state inductions of the nsp13 in complex with the best candidates from both BPP and PP series running explicit-solvent molecular dynamics simulations in three independent replicas each. Additionally we simulated the apo protein as a negative control totaling approximately 300 ns of RMSD-stable production time (Appendix A2) divided in three replicas.

Ligand protonation states were assigned according to the most populated p*K*_a_ species. For MWAC-3963, multiple protonation states are present at physiological pH (Appendix B1), with the +1 species predominating (58.6%). MWAC-3972 exhibits three macroscopic p*K*_a_ values (0.96, 8.70, and 10.93) corresponding to sequential deprotonation of its basic centers (Appendix B2). At pH 7.4, it exists mainly in the doubly protonated form, with minor populations of the +3 and +1 species.

Stable binding modes were subsequently extracted *via* hierarchical agglomerative clustering, and key interactions were characterized using residue-level contact occupancies, geometrically defined hydrogen bonds, and MM/GBSA energies per residue.

#### 2.2.2 BPP: proposed binding mode for MWAC-3972

The helicase (apo state) in complex with MWAC-3972 was solvated using the TIP3P water model in a 0.15 M NaCl environment, was simulated for 1.057 µs. The RMSD traces (Appendix A4) show that replicas 2 and 3 remained close to the initial binding pose during the first 100 ns. Replica 2 subsequently underwent a modest conformational transition of approximately 0.75 Å, converging toward a pose similar to that adopted early by replica 1. Both replicas then maintained stable RMSD values with only minor internal reorientations throughout the remainder of the simulation. In contrast, replica 3 preserved its initial pose for most of the trajectory before exhibiting a more pronounced displacement of approximately 1.75 Å near 225 ns. Although no complete unbinding event was observed, the ligand did not re-stabilize following this transition (Appendix A6).

**Fig. 4:**
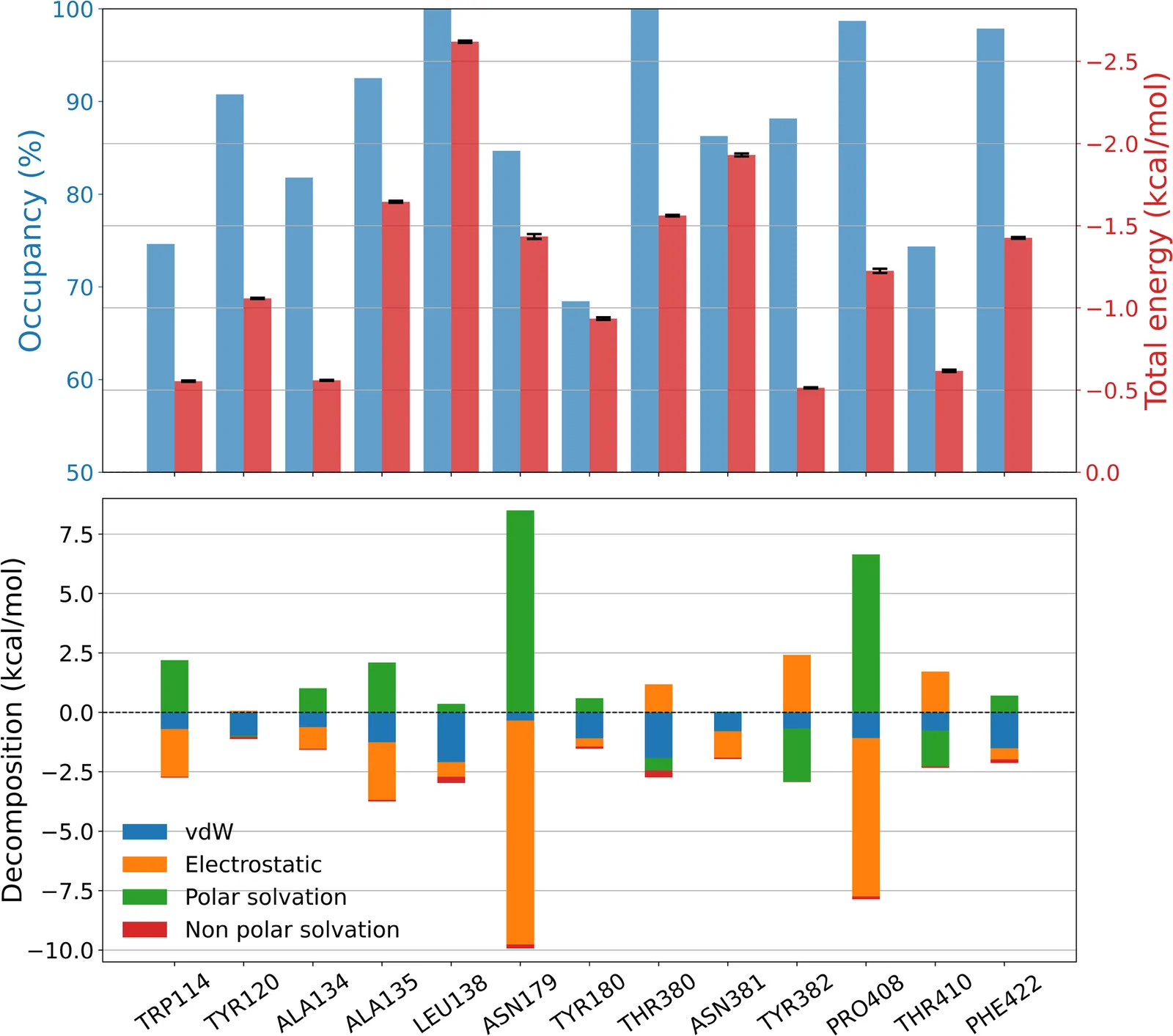
Key-interactions found for MWAC-3972 using contact occupancies, across three replicas of ≈ 350 ns each, and MM/GBSA energies. On top, total energies in kcal mol^−1^ (red) and occupancy across trajectories (blue) are shown side by side for the residues displaying occupancies > 50% and energies < −0.5 kcal mol^−1^. At bottom, the decomposition in vdW (blue), electrostatic (orange), polar (green) and non-polar (red) terms are also plotted.

To characterize binding modes and their occupancies throughout the trajectories, we performed hierarchical clustering of the ligand heavy-atom poses using an *ε* cutoff of 0.8 Å (Appendix C). This analysis identified nine populated clusters with more than one frame (Appendix C1). To identify key interactions, we applied the following criteria: contact occupancies > 50% and MM/GBSA total Δ*G* < – 0.5 kcal mol^−1^ (Fig. 4).

**Fig. 5:**
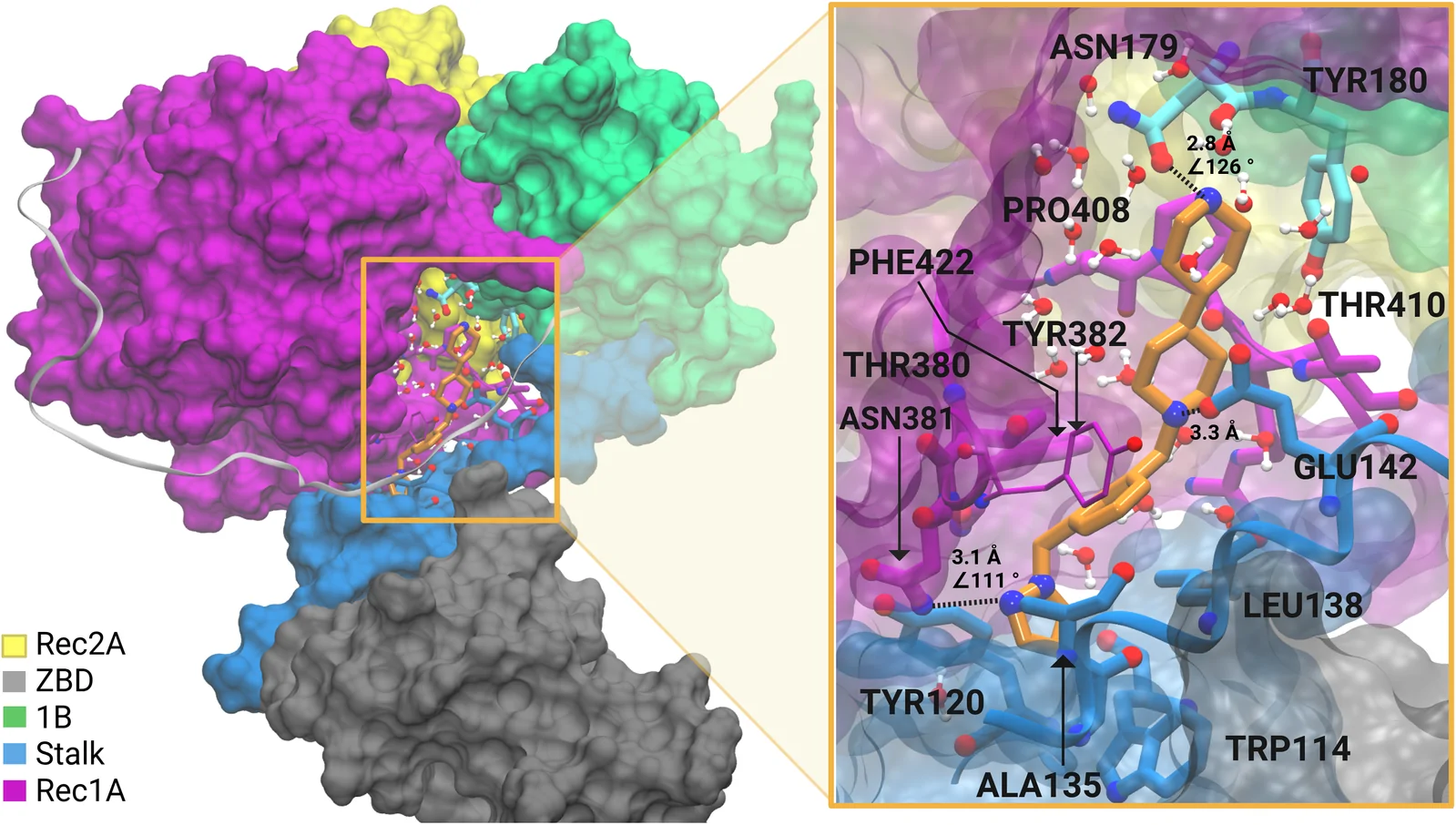
Representative binding mode of MWAC-3972 at the interface between the stalk and Rec1A domains of the target helicase. The overall protein architecture is shown as a surface representation with individual domains colored as follows: Rec1A (magenta), Rec2A (yellow), 1B (green), Stalk (blue), and ZBD (grey). The inset provides a close-up view of the ligand (right), spotlighting key non-covalent interactions that stabilize MWAC-3972 (orange) within the binding cleft.

The most populated cluster, accounting for 67% of the total simulation ensemble (Fig. 5), was used to characterize the spatial organization of the non-bonding interactions.

Across the trajectory, the pyrazole–phenyl moiety of MWAC-3972 engages a cluster of stalk-proximal residues that collectively define a stable hydrophobic anchoring site. The dominant stabilization arises from vdW interactions, including a persistent pyrazole edge-to-face contact with TYR120 and a narrow hydrophobic groove formed by TRP114, ALA134, ALA135, and LEU138. The latter contributes approximately −2.08 ± 0.46 kcal mol^−1^ alone, primarily through favorable packing around the phenyl ring of the ligand. Electrostatic contributions are detected for TRP114 and ALA135 at the backbone, but these favorable terms are largely offset by their corresponding polar solvation penalties.

This behavior is well illustrated by GLU142, whose electrostatic contribution is strongly favorable (−60.6 ± 8.2 kcal mol^−1^), yet the polar solvation term is nearly equal and opposite (+62.7 ± 9.0 kcal mol^−1^). A distance-based analysis across the three trajectories showed that 66.4% of frames exhibited NH⋯ O separations below 3.3 Å, indicating a strong hydrogen-bonding and possible salt-bridge formation. However, this interaction was effectively masked in the MM/GBSA decomposition due to the near-complete cancellation between the electrostatic and polar solvation terms.

The two interacting residues from the protruding loop of the 1B domain are ASN179 and TYR180. ASN179 forms a hydrogen bond with the terminal charged amine of piperidine (NH⋯O = 2.8 Å; ∠C–O⋯*N* = 126.2°), and the adjacent TYR180 stabilizes the pyrazole ring through a persistent T-shaped π – π interaction.

Several interactions involve the Rec1A domain. The strongest occurs with ASN381, where the pyrazole ring forms a weak NH⋯ N hydrogen bond (NH⋯*N* = 3.08 Å; ∠N–H⋯*N* ≈ 111°), complementing hydrophobic contacts with THR380 and PRO408. The BPP moiety extends into a partially open channel between the RecA system and the 1B domain that remains highly hydrated throughout the trajectory. As a result, TYR382 and THR410 contribute mainly through solvent-mediated interactions rather than direct ligand contacts, and PHE422 provides additional stabilization through vdW interactions with the phenyl ring.

Overall, MWAC-3972 adopts a binding mode characterized by a hydrophobic anchoring site at the stalk, selective polar contacts at the 1B loop and Rec1A domain. Moreover, the BPP regions remains highly solvated, reflecting the strong solvent accessibility of this region rather than direct ligand engagement.

#### 2.2.3 PP: proposed binding mode for MWAC-3963

**Fig. 6:**
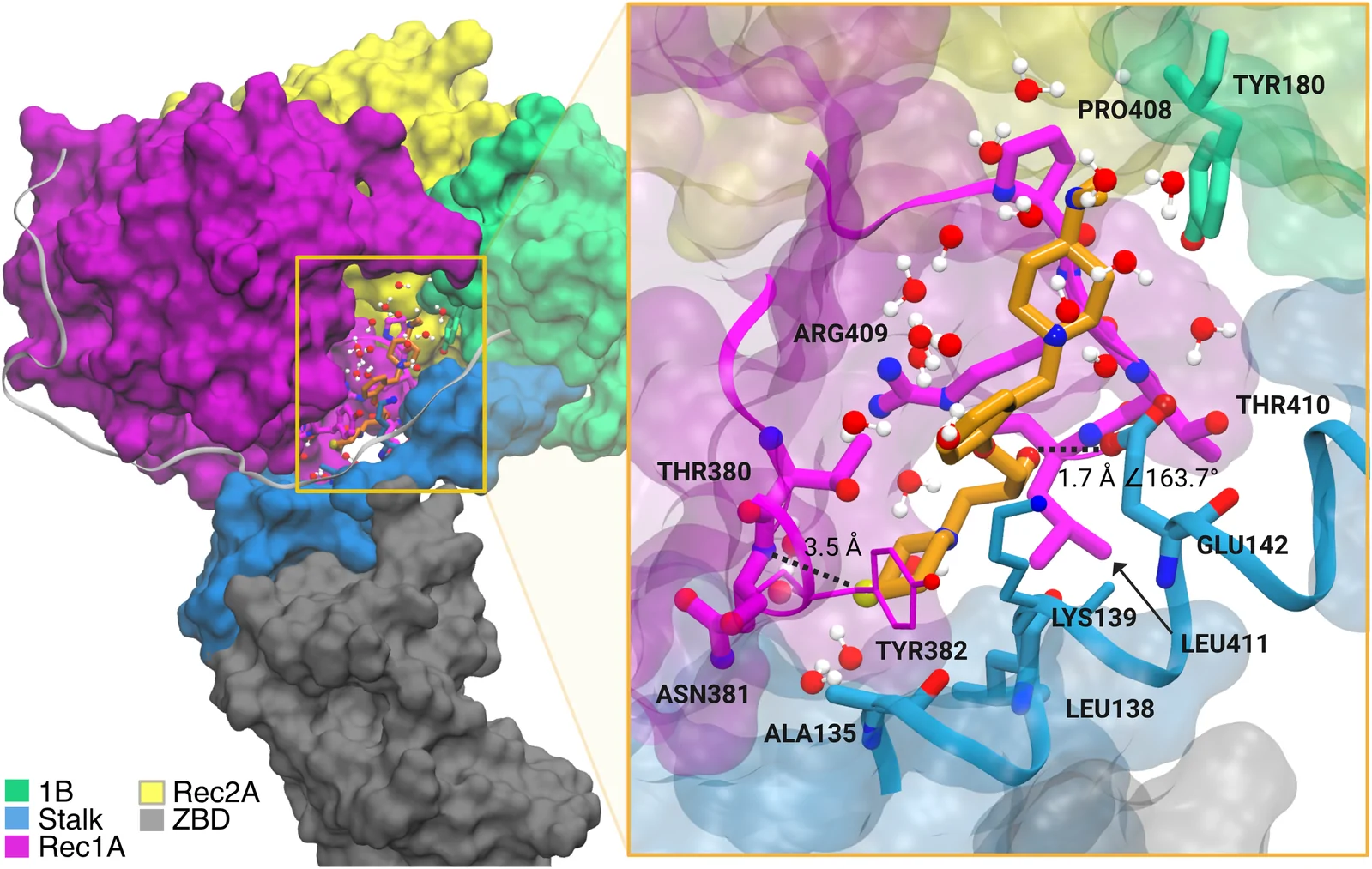
Representative binding mode of the MWAC-3963 across replicas. Protein is colored by domains, ligand is shown in orange licorice and displayed residues correspond to the key-contacts identified through occupancy, MM/GBSA and hydrogen bond formations.

The binding mode of MWAC-3963 was characterized through triplicate molecular dynamics simulations, yielding a total production time of 1.097 µs. The ligand maintained a remarkably stable binding mode throughout the trajectories (Fig. 6, Appendix A3), as confirmed with the hierarchical clustering approach, where only a single cluster was identified and the ligand RMSD presented shifts of maximum 0.65 Å, consistent with rotations and re-orientation within the initial active site (Appendix A5).

The MM/GBSA energy decomposition (Appendix D) indicates that binding is driven primarily by vdW interactions. The engagement mode is similar to that of MWAC-3972, in that both ligands engage longitudinally at the Rec1A–stalk interface, and making only minimal, tip-wise contact with the 1B domain.

The thiazine ring of MWAC-3963 inserts into the stalk domain, forming electrostatic interactions with ALA135 backbone, significant vdW contacts with LEU138 and favorable polar solvation terms with LYS139 and THR141. In particular, LYS139 exerts electrostatic repulsion on the positively charged tertiary ammonium group, although this effect is mitigated by solvation effects. Another remarkable interaction occurs between GLU142 and the hydroxyl group of the ligand. Similar to the MWAC-3972 case, this specific interaction was over-sighted in MM/GBSA total results, due to the polar solvation term. The overestimation is likely because GLU142 is clearly acting as a hydrogen bond acceptor characterized by a highly conserved (100%) contact with the ligand and a distance of OH⋯O = 1.7 Å with an angle of (∠O–H⋯O = 163.7°). Metrics that are compatible with a strong hydrogen bond formation (Fig. 6).

Within the 1B domain, there is a single significant interaction with TYR180, primarily driven by vdW forces and a transient hydrogen bond formation between ASN179 and the charged amino of the ligand, although this interaction was found in only one of the replicas.

In the Rec1A domain, the ligand establishes vdW interactions with THR380, TYR382 and LEU411. A possible hydrogen bond was identified between ASN381 amino backbone and the sulfur of the thiazine. A weak electrostatic interaction is found between PRO408 backbone and nearby methylated amino with an average hydrogen bond formation of 11.7%. ARG409-LEU411 contribute predominantly through water-mediated interactions, with ARG409 and LEU411 additionally providing substantial vdW interactions.

In summary, the binding profile of MWAC-3963 is dominated by a combination of hydrophobic interactions and solvent-modulated polar contacts. In contrast, the MWAC-3972 binding mode exhibits stronger electrostatic interactions, consistent with its modestly improved nLuc EC_50_ potency.

As noted earlier, the polar solvation component should be interpreted with caution, as it typically exhibits the largest deviations when compared with more rigorous reference calculations.^21^ Implicit solvent models tend to underestimate desolvation penalties, particularly for buried charged residues surrounded finely structured water networks. As seen for our GLU142, neglecting these effects leads to exaggerated polar terms that disrupts the natural compensation between solvation and electrostatic terms, ultimately affecting the evaluation of polar residue interactions.^22^ Therefore, relying on favorable polar solvation to rationalize or guide ligand binding optimization is less robust than designing explicit electrostatic interactions that would make sense from a geometrical point of view.

#### 2.2.4 Putative associated mechanisms at an inter-domain level

**Fig. 7:**
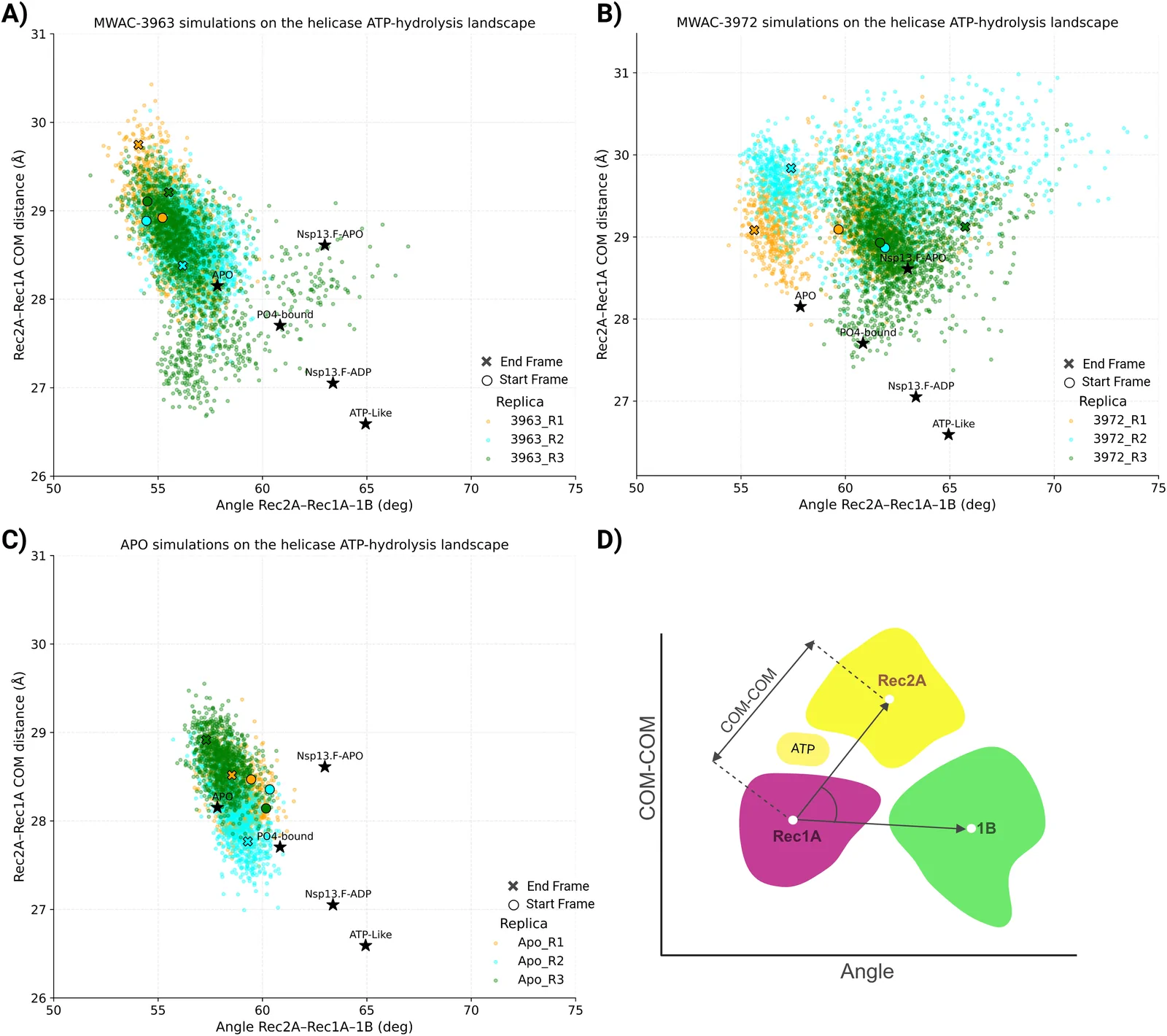
Center Of Mass (COM) inter-domain distances sampled during the MD simulations for (A) MWAC–3963, (B) MWAC–3972, and (C) APO simulations (ligand-free). Each point corresponds to an individual trajectory frame and is colored by replica. (D) Diagram indicating two key nsp13 domain displacements during translocation and used to build the scatter plots. Such displacements were measured by COM distances between domains 1B–Rec1A (*x*-axis) and Rec2A–Rec1A (*y*-axis) and report on global helicase conformational states. Black stars in the scatter plots denote reference distances derived from crystallographic nsp13 structures, including APO (PDB ID 7NIO), nsp13.F–APO (7RDZ: F), phosphate-bound (6ZSL), ATP-like (7NN0), and nsp13.F–ADP (7RDY: F) states.

To elucidate mechanistic differences between the two binding modes, we monitored ATP-dependent interdomain motions central to helicase activity, focusing on Rec1A–Rec2A displacement and 1B domain rotation. The 1B rotation was quantified as the angle defined by the Rec2A–Rec1A–1B centers of mass (COM), and opening of the nucleotide-binding site was tracked by the Rec2A–Rec1A COM separation. The sampled conformational space was contextualized using crystallographic nsp13 structures representing key states across the ATP hydrolysis cycle, including the apo nsp13_F_ state (Fig. 7).

When projected onto this representation, the sampled conformational ensembles define a clearly identifiable inverse relationship between inter-domain separation and 1B rotation, recapitulating the progression of ATP hydrolysis associated nsp13 states. This landscape therefore provides a useful mechanistic framework for interpreting ligand induced modulation of helicase mechanisms of motion.

Within this framework, both ligands sample apo and PO4-bound states while exhibiting increased flexibility in the Rec1A–Rec2A region relative to the apo simulations. However, MWAC-3972 stabilizes a convergent conformational ensemble largely overlapping the nsp13_F_ apo state, whereas MWAC-3963 samples conformations surrounding the isolated apo nsp13 state. Interestingly, one replica transiently accesses shorter Rec2A–Rec1A separations within the range of the ADP-bound reference (closed). However, the narrower interdomain angle is more characteristic of a partially closed intermediate than a canonical ADP-like configuration.

The larger Rec2A–Rec1A–1B angles observed for MWAC-3972 indicate stronger control over 1B domain rotation and arise from several cooperative structural factors. First, MWAC-3972 forms a persistent hydrogen bond with ASN179, whereas MWAC-3963 engages this residue only transiently. Second, GLU142 interacts strongly with both ligands but adopts different orientations. In the MWAC-3972 complex, it points toward the solvent-exposed entry and disrupts a transient native hydrogen bond with THR410. In MWAC-3963, this bond is maintained because the ligand hydroxyl group stabilizes GLU142. In MWAC-3972, the outward orientation of GLU142 is accompanied by displacement of the extended stalk α-helix, directly linked to the 1B domain. Further differences occur in the flexible loop (residues 400–418), where ARG409 moves away from MWAC-3972, possibly due to electrostatic repulsion from its two positively charged amino groups.

Taken together, these observations suggest that the stronger effect of MWAC-3972 in the replication assay relative to MWAC-3963 arises from preferential stabilization of an apo-like nsp13_F_ conformational ensemble. MWAC-3972 also exerts stronger control over 1B domain rotation through interactions with ASN179 and TYR180, thereby biasing the helicase toward open-trapped states. In this view, modulation of 1B dynamics is not an alternative mechanism but a direct consequence of stabilizing the apo state.

### 2.3 Structure–activity relationship trends in the two hit series

We performed an R-group decomposition of the two lead series to determine which scaffold positions are crucial for activity and to identify specific substituents that improve binding affinity or reduce cytotoxicity.

**Fig. 8:**
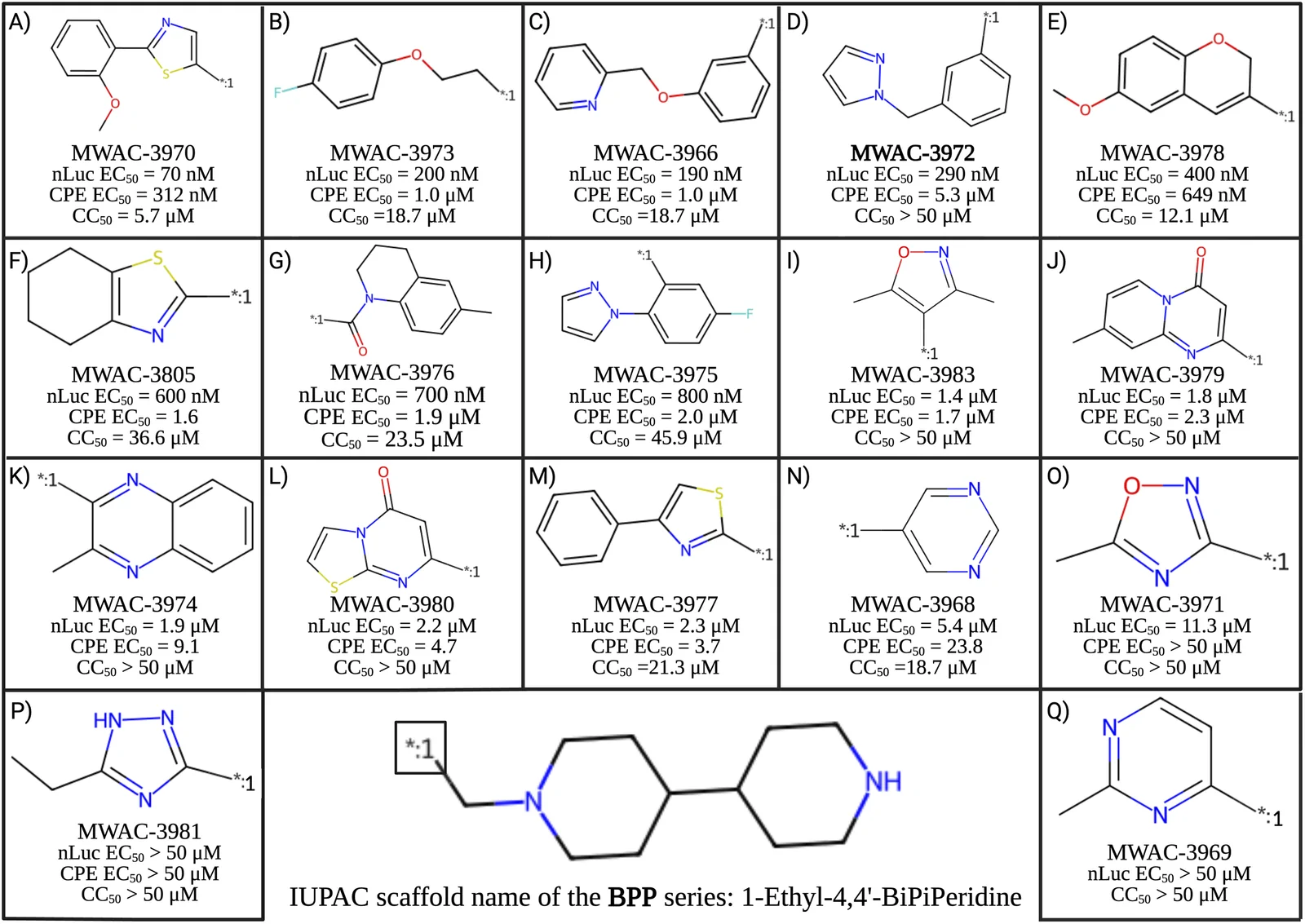
R-Group decomposition of the MWAC-3805 scaffold series. Substitutions at position *:1 are shown (A–Q), with corresponding MWAC identifiers and antiviral assay data (nLuc EC_50_, CPE EC_50_, and CC_50_). Several analogs demonstrate submicromolar activity (*e.g.*, MWAC-:3966, 3970, 3972, 3973, 3978, 3805, 3976, 3976), though the differences in cytotoxicity underline the importance of scaffold tuning.

The analysis of the decomposition for the BPP scaffold (Fig. 8) allowed us to identify substitutions at the R-ethyl-4-4’-BPP position (*:1) that modulate potency and cytotoxicity. Adding anisole substituents increases potency (MWAC-: 3970, 3966 and 3978) at the cost of cytotoxicity, possibly due to metabolic stability issues associated to CYP-mediated *O*-dealkylation. In contrast, aza-aryl containing systems such as MWAC-3968, -3974 and −3971 showed reduced potency relative to MWAC-3805. Among the BPP analogs, MWAC-3972 emerged as the most favorable compound in terms of both potency and cytotoxicity. Similar to MWAC-3975 and MWAC-3966, it contains a methyl-substituted benzene ring adjacent to the *:1 position, pointing to a potential structural feature for optimizing the therapeutic index within this sub-series. MWAC-3983 is safe in terms of cytotoxicity and performed similarly as MWAC-3805 with low-micromolar potency in both CPE and nLuc assays. Finally, only one substitution at the secondary amine of the BPP was tested (MWAC-3965) but it did not produce any detectable measure in potency, hence is not present in this figure.

**Fig. 9:**
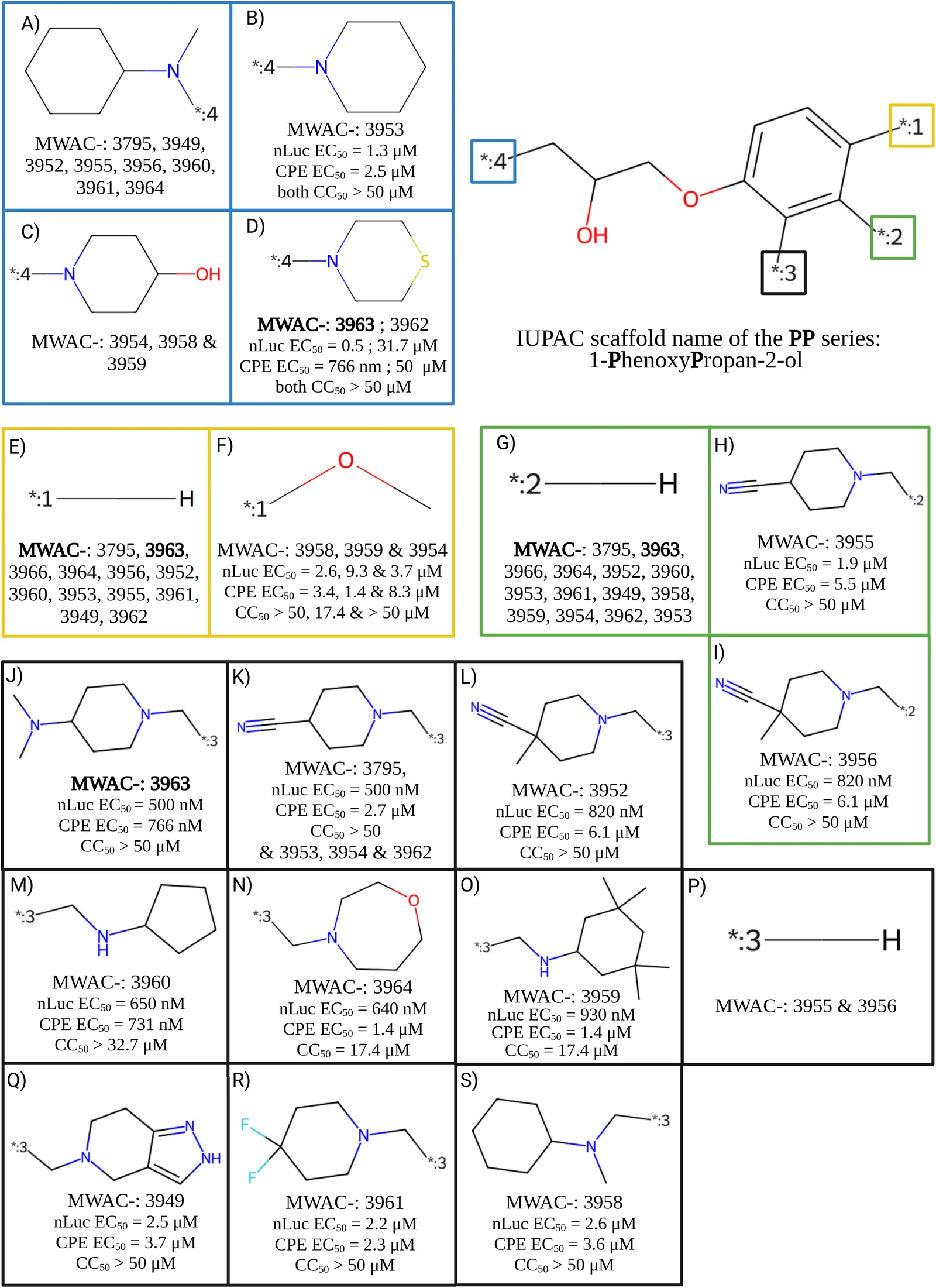
R-Group decomposition of the PP series. The central scaffold corresponds to 1-phenoxypropan-2-ol, with positions of substitution marked (*:1–*:4). Representative R-group variations at these positions (A–S) are shown together with associated MWAC identifiers and antiviral assay results (nLuc and CPE EC_50_, CC_50_).

The 1-ethylpiperidine-4-carbonitrile of the PP series (Fig. 9), present in MWAC-3795, loses activity when moved from the third to the second position (MWAC-3955). This substitution results in a fourfold decrease in potency in the viral replication assay and a twofold decrease in the cytopathic effect assay, underscoring the third position as crucial for potency modulation.

At the third position, substitution of the nitrile group with a dimethylated amino moiety in MWAC-3963 improved protection against virus-induced cytopathic effects while maintaining potent replication inhibition relative to MWAC-3795. This modification appears to strongly influence antiviral activity, as these compounds differ only at the third and fourth positions. However, the structure–activity relationship is not straightforward: MWAC-3963 and MWAC-3962 share the same fourth-position substituent yet differ substantially in potency, while MWAC-3962 and MWAC-3795 share the same third-position substitution. Since all three compounds contain identical substituents at the first two positions, the results suggest that activity is governed by the combined effects of substitutions at the third and fourth positions.

### 2.4 Drug-like profiles for the two hit series

To evaluate the developability of our lead compounds, we obtained a set of molecular descriptors commonly associated with oral drug-like properties from the CDD Vault^23^ and summarized in Table 3. When these descriptors fall within balanced physicochemical ranges, they are generally associated with favorable drug-like behavior, including reduced molecular weight (≤ 500 g mol^−1^), improved aqueous solubility (log *S* between −5 and −1), moderate lipophilicity conducive to membrane permeability (log *P* ∼1–3 (ref. 24)), and balanced partitioning of ionized and non-ionized species at physiological pH (log *D*_7.4_ ∼ 1–3 (ref. 25)). Additional properties associated with oral bioavailability include a Topological Polar Surface Area (TPSA) ≤140 Å^2^,^26^ approximately six rotatable bonds,^26^ and Fsp^3^ values, >0.42 (ref. 27) associated with improved binding specificity and reduced promiscuity due to increased molecular three-dimensionality.

**Table 3:** Molecular properties of selected compounds from the PP and BPP series

Molecule	Series	MW (g mol^−1^)	log *P*	log *D*	log *S*	TPSA (Å^2^)	Fsp^3^	Rot. bonds
MWAC-3963	PP	393.6	2.4	0.3	−4.2	39.2	0.71	8
MWAC-3795	PP	385.6	3.2	1.6	−4.7	59.7	0.70	8
MWAC-3960	PP	360.5	4.0	1.6	−5.1	44.7	0.73	9
MWAC-3964	PP	376.5	2.7	1.4	−4.3	45.2	0.73	8
MWAC-3952	PP	399.6	3.5	1.3	−5.0	59.7	0.71	8
MWAC-3961	PP	396.5	3.5	2.1	−5.0	35.9	0.73	8
MWAC-3953	PP	357.5	3.0	1.4	−4.4	59.7	0.67	7
MWAC-3983	BPP	277.4	1.6	0.6	−2.9	41.3	0.81	3
MWAC-3972	BPP	338.5	1.9	1.0	−3.6	33.1	0.57	5
MWAC-3805	BPP	319.5	2.9	2.0	−4.1	28.2	0.83	3
MWAC-3975	BPP	342.5	2.6	1.4	−4.1	33.1	0.55	4
MWAC-3979	BPP	340.5	1.2	0.8	−3.0	49.6	0.60	3

Both the BPP and PP series comply with Lipinski's rule-of-five criteria. However, the two series differ in key physicochemical properties, particularly molecular weight and TPSA, with the BPP series exhibiting lower values that may provide greater flexibility for lead optimization. Also, half of the PP molecules display limited aqueous solubility (log *P* values > 3), potentially reducing absorption.^28^ Noteworthy, the BPP series show lower count of rotatable bonds (avg. 4) than the PP (avg. 8) implying lower entropy costs when binding to nsp13 or crossing cellular membranes, although both series have acceptable flexibility for oral bio-availability.^26^

The Fsp^3^ values for both series (≥0.60) are above the median value reported for most approved drugs (0.42).^29^ This high degree of saturation suggests that the compounds are more three-dimensional and less planar, reducing the likelihood of non-specific binding and pan-assay interference.

Consistent with the physicochemical profiles, metabolism assessed *via* CYP450 inhibition measures against the four major isoforms (CYP1A2, CYP2C9, CYP2D6, and CYP3A4) revealed clear series-dependent trends (Table 4). Most compounds exhibited ≤50% inhibition at 10 µM, except for PP analogs MWAC-3964, -3960, and -3952, which strongly inhibited CYP2D6 (> 80%). Importantly, none of the tested compounds showed significant inhibition of CYP3A4, the isoform most commonly associated with clinical drug–drug interactions.^30^

**Table 4:** Cytochrome P450 inhibition profile for selected PP and BPP compounds. Values are reported as percent inhibition

Compound ID	Series	CYP1A2	CYP2C9	CYP2D6	CYP3A4
Furafylline (40 µM)	Ctrl	91	<10	<10	38
Sulfaphenazole	Ctrl	31	84	<10	47
Quinidine	Ctrl	27	<10	83	30
Ketoconazole (1 µM)	Ctrl	<10	<10	46	97
MWAC-3983	BPP	20	10	31	18
MWAC-3972	BPP	11	<10	44	18
MWAC-3805	BPP	18	<10	10	18
MWAC-3964	PP	15	10	**80**	40
MWAC-3963	PP	14	10	28	31
MWAC-3960	PP	26	10	**81**	42
MWAC-3952	PP	32	10	**85**	41

To assess metabolic stability, microsomal stability assays were performed in human and mouse liver microsomes (0.5 mg mL^−1^ protein) for representative members of both series (Table 5). The results from both assays are consistent with one another: BPP analogs showed minimal CYP inhibition, exhibited excellent microsomal stability (*t*_1/2_ > 120 min), and had low intrinsic clearance (Cl_int_ < 10 µL min^−1^ mg^−1^). Regarding PP analogs, they displayed strong CYP2D6 inhibition (≥80%) and markedly higher intrinsic clearance values, particularly in mouse microsomes.

**Table 5:** Microsomal stability (*t*_1/2_, minutes) and intrinsic clearance (Cl_int_, µl min^−1^ mg^−1^) of MWAC compounds compared with sunitinib, with series annotation

Compound ID	Series	*t*_1/2_ (min)		Cl_int_ (µl min^−1^ mg^−1^)	
		Human	Mouse	Human	Mouse
Sunitinib	Control	31.8	24.6	44	56
MWAC-3983	BPP	>120	>120	< 10	< 10
MWAC-3972	BPP	>120	>120	< 10	< 10
MWAC-3805	BPP	>120	15.0	< 10	92
MWAC-3964	PP	49.1	5.3	28	261
MWAC-3963	PP	104.6	7.1	13	194
MWAC-3960	PP	42.3	6.1	33	226
MWAC-3952	PP	13.4	4.6	104	301

Although the BPP analogs display favorable microsomal stability and drug-like physicochemical descriptors, the cationic nature of both scaffold classes under physiological pH may introduce additional developability considerations beyond hepatic metabolism. Protonatable amines can influence passive membrane permeability and may promote off-target liabilities such as hERG channel interactions or/and recognition by efflux transporters such as P-gp and BCRP, potentially limiting intracellular exposure or oral absorption.^31^

In addition, the two ionizable amine functionalities in BPP and some amino groups in samples of the PP series, such as MWAC-3963 and -3795, contain multiple protonatable amines with calculated basic p*K*_a_ values ≥6 (ref. 32) and log *P* values above 1, features associated with lysosomal trapping.^33^ However, many analogs from both series, including MWAC-3963, showed cytotoxicity values above 50 µM in A549-hACE2 cells, supporting limited overt viability loss under the assay conditions.

These metabolic results align with the physicochemical characteristics of each series. The BPP analogs show drug-like solubilities (log *S* between −4 and −3), balanced lipophilicity (log *P* and log *D* in the 1–3 range), and fewer rotatable bonds (approximately four per molecule). Together, these features favor good oral bioavailability, low nonspecific binding, and reduced entropic penalties upon enzyme binding. Notably, MWAC-3983 displays higher solubility than the other analogs and may increase the unbound microsomal fraction, while still retaining a low Cl_int_ pointing to limited metabolic clearance.

By contrast, several PP analogs lie near the limits of the developability window. MWAC-3960 and -3952 combine low solubility (log *S* ≤ −5.0) with high flexibility (8–9 rotatable bonds), properties that may increase productive binding within CYP active sites and expose oxidizable α-carbons adjacent to basic nitrogens. In accordance with this, MWAC-3964, -3960, and -3952, all containing a tertiary amino substituent at the R4 position of the PP scaffold (Fig. 9), exhibited strong CYP2D6 inhibition, in agreement with the reported *N*-dealkylation of tertiary amines by this enzyme.^34^

Among the PP series, MWAC-3963 stands out as the most balanced candidate. Its very low log *D* (0.3) reflects high polarity and limited passive permeability, yet this property probably contributes to its moderate intrinsic clearance and acceptable half-life in human microsomes. Considering its p*K*_a_ under a neutral pH, the positive charge of this compound might limit its access to the hydrophobic pockets of CYP, accounting for its slower oxidation and explaining its strong antiviral effectiveness against SARS-CoV-2.

Hence, to further optimize the most promising PP analogs, led by MWAC-: 3963, 3795, and 3952, scaffold modifications should aim to (i) raise solubility above approximately 100 µM (log *S* > −4), (ii) reduce the number of rotatable bonds, and (iii) block α-carbon oxidation adjacent to the R4 tertiary amine.

Overall, MWAC-3972 emerged as the most promising compound in terms of potency and predicted metabolic and excretion properties, followed by MWAC-3983 and, to a more conditional extent, MWAC-3805. Their balanced lipophilicity and low intrinsic clearance suggest the potential for improved hepatic exposure at comparable doses. Nevertheless, the cationic character of several hits at neutral pH warrants further investigation, as it could influence interactions with efflux transporters (*e.g.*, P-gp and BCRP), cardiac hERG channels,^31^ and increase the risk of lysosomal trapping.

### 2.5 Proposed structural optimization

Taken together, the interaction analysis, ligand-induced conformational dynamics, and physicochemical profiling converge to define clear design principles for structural optimization of the most promising candidates. These results identify scaffold regions essential for binding, interactions that can be strengthened to enhance conformational control, and chemical features that could limit drug-like behavior. Moreover, within these boundaries, residue-resolved electrostatic analyses guide the introduction of polar functionalities to improve local complementarity. Because electrostatic interactions dominate biomolecular recognition under physiological conditions,^35^ distance-based electrostatic mapping identifies scaffold positions where polar substitutions could provide favorable energetic gains.

**Fig. 10:**
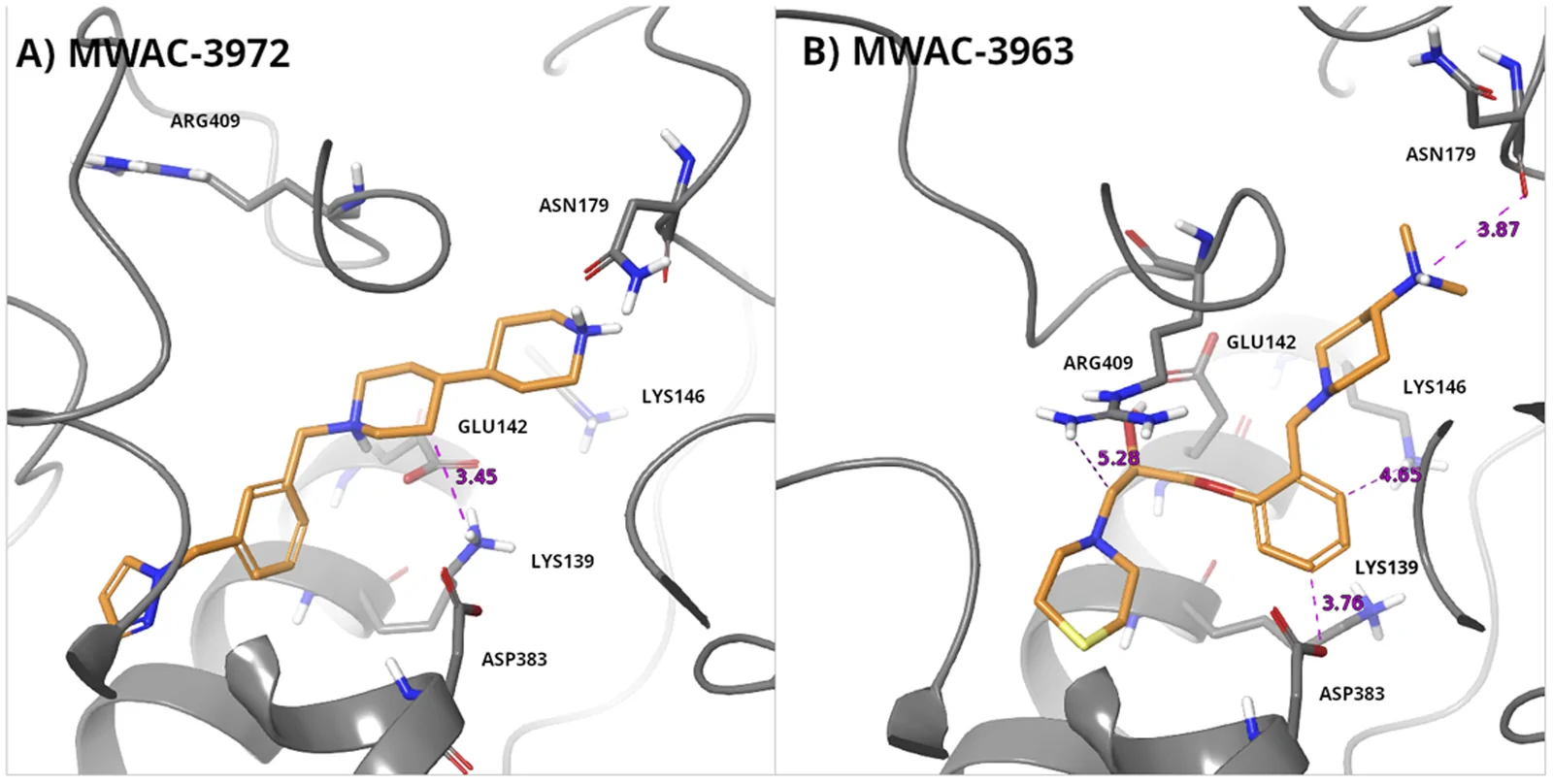
0Protein (gray ribbons) with aligned representative binding poses of (A) MWAC-3972 and (B) MWAC-3963, both shown in orange. Licorice-rendered residues denote regions of strong (*e.g.*, GLU142 and ASN179) or repulsive electrostatic interactions, providing guidance on what molecular regions should be retained or modified during optimization.

For MWAC-3963, replacing the carbon atom at the fifth position of the aromatic ring with a ring nitrogen to generate an aza analogue would introduce a hydrogen bond acceptor oriented toward ASP383 (Fig. 10). This residue is currently positioned at 3.76 Å and contributes only weak stabilization in the parent compound, implying that this modification could convert a suboptimal interaction into a productive contact. In a complementary strategy, a hydroxyl substituent at the fourth position of the same ring could form a hydrogen bond with LYS146, reducing the unfavorable electrostatic contribution of (21 ± 2) kcal mol^−1^. The terminal thioether moiety of MWAC-3963 engages only weakly with the binding pocket and represents a metabolic liability due to its susceptibility to oxidative metabolism, making it a suitable site for replacement. This region can accommodate larger substituents, as evidenced by the deeply buried pyrazole moiety observed in MWAC-3972. In addition, substitution at the thiazine *α* position to promote a directed interaction with ARG409, for example by displacing the adjacent hydroxyl group, could alleviate the existing repulsive contribution of approximately 4.1 kcal mol^−1^. As indicated by MWAC-3972, -3975, and -3966, introduction of a biaryl shaped substituent at the PP R3 position could further enhance potency by increasing molecular extension and promoting anchoring interactions with the stalk region, thereby reinforcing contacts with the 1B domain. Finally, although both ligands form strong hydrogen bonds with GLU142, the geometry and donor characteristics differ substantially. The charged amine of MWAC-3972 mediates a significantly stronger electrostatic interaction, as reflected by MM/GBSA estimates of −60.61 ± 8.19 kcal mol^−1^ compared to −30.94 ± 2.5 kcal mol^−1^ for MWAC-3963, pointing to a replacement of the hydroxyl group with a cationic donor as a more favorable design strategy.

In MWAC-3972, the terminal amine forms a favorable interaction with ASN179; however, this contact is unstable and is lost in one replica as ASN179 reorients toward ASP534, resulting in destabilization of the binding mode. By contrast, the dimethylamino group of MWAC-3963 does not meaningfully engage ASN179. These observations suggest that more optimal substituents could be identified for both scaffolds to strengthen interactions in this region. Specifically, introduction of a carbonyl substituent at the 2 position of the piperidine ring in MWAC-3972 would generate a neutral hydrogen bond acceptor oriented toward LYS139, a residue that currently contributes a highly unfavorable electrostatic term of (46 ± 5) kcal mol^−1^. Such a modification might reduce repulsion and improve local electrostatic complementarity.

Beyond individual contacts, residues spanning ARG178 to TYR180 have been implicated in mediating conformational control of the 1B domain, together with additional residues identified here, including PRO408.^36^ These residues are conserved across the coronavirus family and participate directly in nucleotide engagement. Stabilizing ligand interactions with this flexible 1B loop is therefore expected to interfere with helicase function by restricting the conformational transitions required for productive unwinding.

## 3 Limitations

Our simulations did not include the full RTC, but rather focused on isolated nsp13 or partial assemblies. As a result, long-range allosteric effects, cooperative interactions between RTC components, and steric constraints imposed by the full complex were not explicitly modeled. This simplification might influence both binding modes and the functional consequences of ligand engagement in the cellular context.

Second, we observed a discrepancy between cell-based antiviral activity and biochemical unwinding assays, as different compounds measurably inhibited helicase unwinding under the experimental conditions employed, compared to the candidates identified in the nLuc and CPE assays. Our interpretation invoking preferential engagement of a second nsp13 copy within the RTC assumes that both assay modalities faithfully report on nsp13 function. However, this assumption does not account for assay-specific artifacts, differences in sensitivity, or context-dependent requirements for inhibition. As such, the proposed mechanism should be considered a working hypothesis rather than a definitive explanation. However, biophysical or crystallographic experiments such as SPR or cryo-EM are required to confirm nsp13 specificity.

Finally, while the identified compounds demonstrate activity compatible with perturbation of nsp13-dependent processes, we cannot exclude the possibility that their antiviral effects arise, at least in part, from interactions with other components of the RTC or even viral structural proteins. Off-target engagement within the viral replication machinery remains a plausible alternative explanation, particularly in the context of cell-based assays where multiple viral and host factors contribute to the observed phenotype.

## 4 Conclusion

This work integrated a MPNN, molecular docking, MM/GBSA re-scoring, and structural clustering to prioritize a highly focused set of purchasable compounds for experimental evaluation against SARS-CoV-2. In total, 75 compounds were experimentally evaluated, with 60 yielding complete and valid measurements across nLuc, CPE, and cytotoxicity assays in A549-hACE2 cells. We achieved a hit rate of 52%, attributed in part to the availability of prior experimental data combining both enzymatic and cell-based readouts during model training, biasing the model toward features associated with intracellular target engagement. This interpretation is further supported by the absence of statistically significant correlations between docking-based metrics and the experimental activity measurements.

Across the best candidates, we identified two distinct inhibitor series: the BPP series, representing a previously unreported scaffold, and the PP series, derived from compounds previously identified by our group. Notably, both series include nanomolar-potent inhibitors. Regarding ADME profiles, the BPP series exhibited more favorable metabolic and excretion descriptors, as evidenced by CYP450 and liver microsome stability assays, supporting an intrinsically safer scaffold. In contrast, the PP series would benefit from improvements in solubility, targeting log *S* values above −4, and from reducing molecular flexibility, ideally limiting the number of rotatable bonds to < 4.

From a deep-learning perspective, the identification of the BPP scaffold as a novel chemotype, together with the strong AUROC performance observed in the blind test set, demonstrate the extrapolative capacity of the UMPNN model beyond close structural analogues. Additionally, MM/GBSA can misrepresent key polar interactions, masking important electrostatic effects. We therefore recommend complementing energetic analyses with explicit geometric measurements to more accurately capture solvation-sensitive contacts. Although experimental validation of direct nsp13 targeting is still lacking, we hypothesize that these compounds act as helicase inhibitors, as the training data were predominantly derived from nsp13 unwinding inhibition assays and the candidates displayed stable binding across three independent molecular dynamics simulations. These simulations revealed ligand-induced conformational changes in agreement with either RNA-interfacial inhibition or stabilization of a pre-hydrolytic state in which the THIQ pocket remains accessible. In particular, MWAC-3963 binding appeared to be driven mainly by hydrophobic interactions combined with solvent-modulated polar contacts, whereas MWAC-3972 exhibited stronger electrostatic contributions, explaining its approximately one-fold improved potency in the nLuc assay.

Furthermore, the interaction differences between the two simulated candidates were also reflected at the conformational level. Both ligands promoted apo-trapped states of nsp13 through modulation of the 1B domain, as supported by multi-state molecular docking, molecular dynamics simulations, and -R-group decomposition analyses, where direct interactions (fourth position) with the 1B domain appeared to influence antiviral potency in the case of MWAC-3963. However, conformational MD analysis suggested that MWAC-3972 was better able to engage the second nsp13_F_ subunit within the RTC complex, due to stronger stabilization and control of the 1B domain.

Accordingly, further optimization of the PP series, particularly for compounds such as MWAC-3963, should draw inspiration from structural features present in MWAC-3972, designing substituents that promote tighter RecA1-1B coupling. Additionally, modifications could lookout to mitigate metabolic liabilities associated with thiazoline motifs and α-carbon oxidation.

The main concern with the BPP series is its doubly cationic character under physiological conditions, potentially increasing susceptibility to efflux transporters and hERG-related off-target liabilities. Future optimization efforts could therefore focus on modifying the central amino group, as no direct interactions involving this moiety were observed in our simulations.

## Author contributions

ACCL wrote the manuscript and conducted the computational component of the drug discovery campaign under the guidance and supervision of REA. TDB performed the analog search within the virtual screening libraries and provided medicinal chemistry insights on the selected candidates. REA supplied the computational infrastructure and oversight necessary to execute the simulations. EK carried out all experimental assays under the guidance and supervision of DC. All authors contributed to the discussion of results and approved the final version of the manuscript.

## Conflicts of interest

There are no conflicts to declare.

## Supplementary Material

SC-OLF-D6SC02048H-s001

## Data Availability

The required to reproduce the results of this study are freely available. Supplementary information (SI): detailed computational and experimental methodologies are provided in the Experimental section of the Supporting Information, together with additional ref. 37–47. Additional datasets and methodological details are included in Appendices A–D. See DOI: https://doi.org/10.1039/d6sc02048h. Code and ML datasets: the complete repository required to reproduce the UMPNN model is available at https://github.com/chemdesign-accl/UMPNN (MIT license). The curated training dataset is named dataset.csv Simulation files and analysis scripts: input files and scripts required to reproduce the molecular simulations are provided in the compressed folder Simulations.zip. These include occupancy contact analysis scripts, MMPBSA input files for AMBER, and the parameter and coordinate files used for the simulated systems. Experimental screening results: all experimentally tested compounds and their outcomes are provided in the file total_tested.csv.
