# Supplementary material for: Discovery of two novel small-molecule series with potent SARS-CoV-2 inhibition via putative nsp13 open-state trapping
Source: Chem Sci. 2026 Aug 28;17(40):19342–58. doi: 10.1039/d6sc02048h (PMC13523015; doi:10.1039/d6sc02048h)
Supplement: Supplementary file 1 [file SC-017-D6SC02048H-s001.pdf]

Supplementary Information (SI) for Chemical Science.
This journal is © The Royal Society of Chemistry 2026

Supporting Information

Discovery of Two Novel Small-Molecule Series with Potent SARS-CoV-2 Inhibition via Putative nsp13 Open-State Trapping

Alma C. Castañeda-Leautaud[a], Thomas D. Bannister[b], Eunjung Kim[c], Donghoon Chung[c] and Rommie E. Amaro[a†]

[a] Department of Chemistry and Biochemistry, University of California San Diego, La Jolla, CA 92093-0533, USA.
[b] Department of Molecular Medicine, The Herbert Wertheim UF Scripps Institute for Biomedical Innovation and Technology, Jupiter, FL 33458, USA.
[c] Department of Microbiology and Immunology, University of Louisville, Louisville, KY 40292, USA.
[†] Corresponding author. Email: ramaro@ucsd.edu; Tel: +1 (858) 534-9629.

Table of Contents

1. Experimental....... 2
1.1. Graph Neural Network model for compound prioritization....... 2
1.2. Virtual screening protocol....... 5
1.3. Apo and ligand-bound simulations of SARS-CoV-2 nsp13....... 6
1.4. Non-bonding interactions analysis....... 7
1.5. NanoLuciferase reporter assay....... 8
1.6. Cytotoxicity and viability assays in A549-hACE2 and Vero76 cells....... 9
1.7. nsp13 dsRNA unwinding assay....... 9
1.8. SARS-CoV-2 cytopathic assays....... 10
1.9. Liver Microsomal Stability (LMS)....... 11
1.10. Cytochrome P450 inhibition assay....... 11
References....... 12
Appendix B. Protonation states for MWAC-3963 and MWAC-3972....... 16
Appendix C. Clustering method for obtaining representatives poses....... 17
Appendix D. MM/GBSA and occupancy decomposition for the MWAC-3963 complex....... 19

1. Experimental

1.1. Graph Neural Network model for compound prioritization

We implemented and trained an undirected message-passing neural network. The model processes previously batch-normalized features using multilayer perceptrons (MLPs), whose composition is illustrated in **Fig. 1.1**. Message-passing is formulated as a sequential composition of three blocks (edge, node, and global), following the MetaLayer framework[12]. In the edge block, messages are constructed by generating relational embeddings from the interacting node features X_i and X_j together with their associated edge features e_{ij} and e_{ji}. In the node block, each source node is re-evaluated in the context of incoming messages, and representative embeddings are extracted via max-pooling and aggregated with neighboring nodes in a single pass. In the global block, node level information is lifted to the molecular level by extracting the maximal signal across nodes within each molecule, after which global molecular features are incorporated and processed through a final MLP to produce a single prediction per molecule. Training is performed using binary cross entropy with logits loss (BCE with LogitsLoss) and the Adam optimizer. Model outputs > 0.5 are assigned to class 1, while values < 0.5 are assigned to class 0.

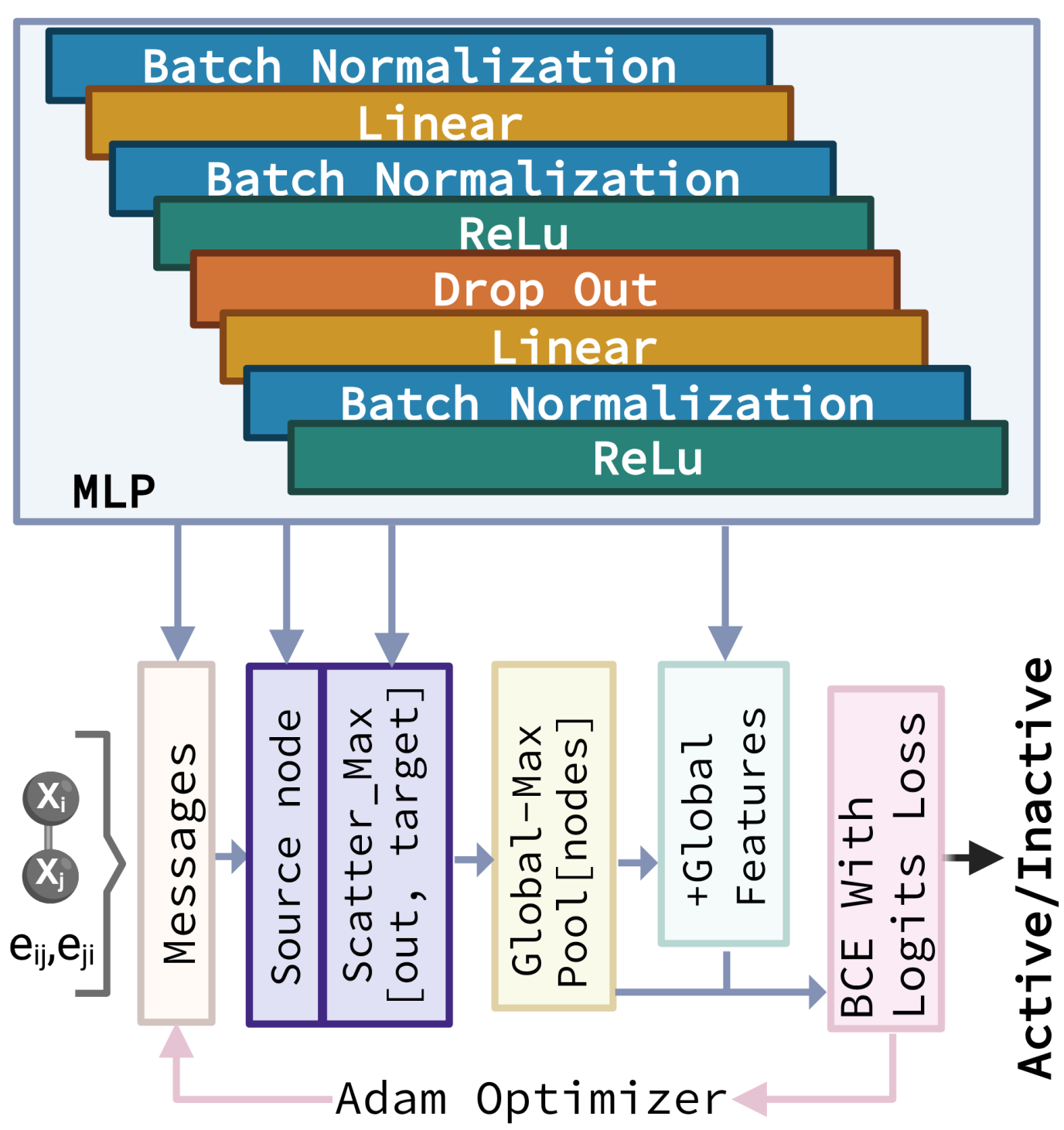

Fig. 1.1. Diagram of our UMPNN architecture for molecular classification. The message is composed of the source (x_i), destination (x_j) nodes, and edge attributes (e_{ij}, e_{ji}). Each gray arrow represents a Multi-Layer Perceptron (MLP), detailed in the top inset, acting on the specified level.

Molecular graphs were represented with a symmetric adjacency matrix where both connectivity and edge feature vectors are duplicated ($e_{ij} = e_{ji}$) to ensure consistent bidirectional flow. Atom, bond, and molecular-level features were extracted and normalized using min–max scaling, with categorical features explicitly mapped to values between 0 and 1 (**Table 1.1**). Feature selection was explicitly optimized to reduce redundancy; within each feature category, candidate descriptors were evaluated, and only those exhibiting pairwise correlations < 0.85 within each graph category were retained (**Fig. 1.2**). Additionally, 3D descriptors were employed based on their demonstrated advantages in undirected message-passing models[12]. Prior to feature extraction, molecular coordinates were optimized using the Merck Molecular Force Field (MMFF), with hydrogens subsequently removed to minimize data dilution.

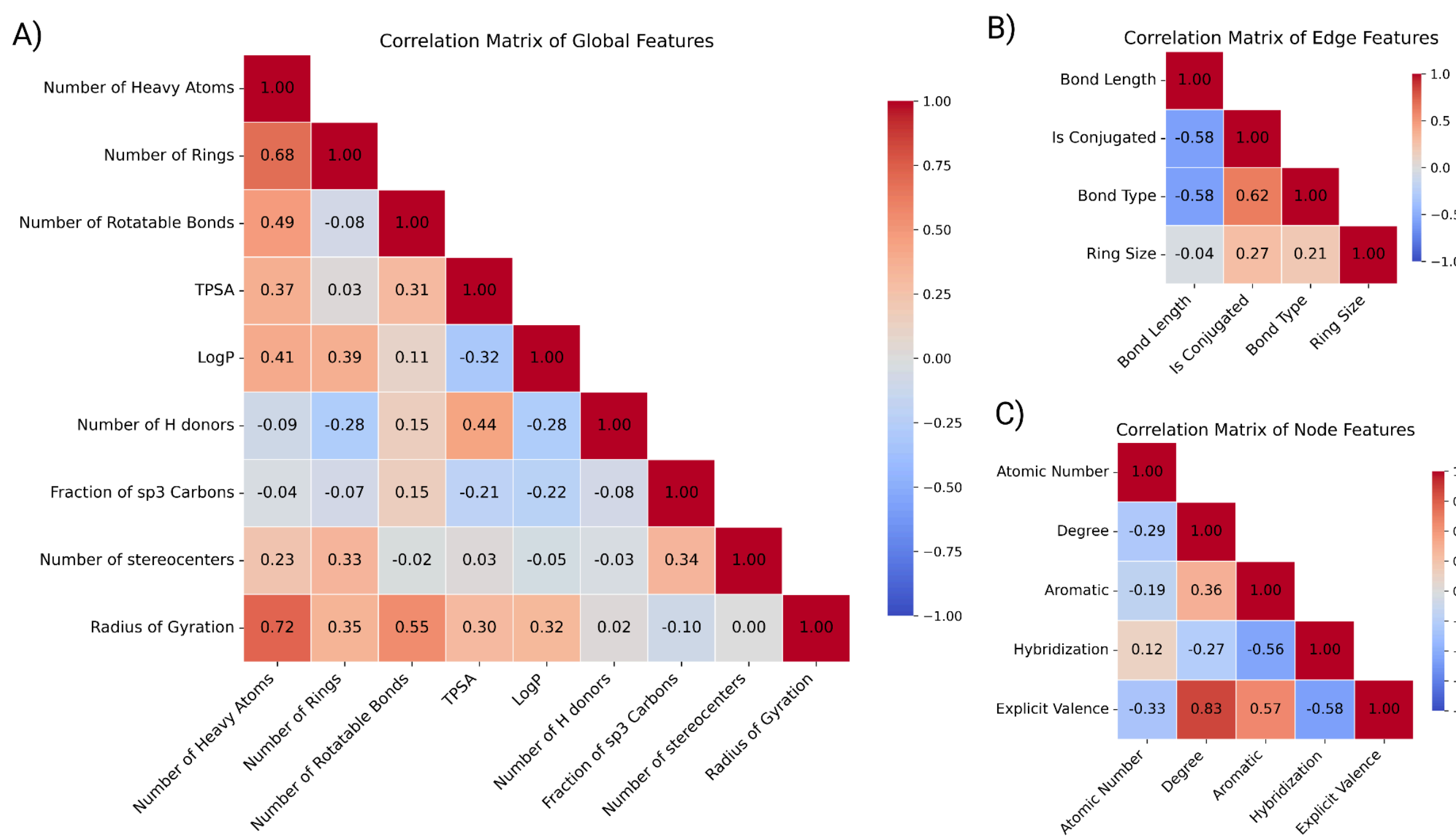

Fig. 1.2. Heatmaps of correlation for global, node, and edge features. Features were selected such that pairwise correlations within each graph category remained < 0.85. (A) Final set of global features, where the strongest retained correlation is between the number of heavy atoms and the radius of gyration. (B) Selected set Edge-level features. (C) List of Node-level features.

The continuous descriptors were min-max normalized, while categorical ones were mapped to values between 0 and 1, the final list of chosen features along with the normalization strategy is summarized in **Table 1.1.**

Table 1.1. Global-, atom-, and bond-level features used in the UMPNN model. Explicit categorical features were dictionary-mapped to values between 0 and 1, while continuous descriptors were min--max normalized.

ID	Feature Type	Encoding / Normalization
**Global Features**		
1	Num Heavy Atoms	Min-Max
2	Ring Count	Value / 6
3	Num Stereocenters	Value / 5
4	Num H-bond Donors	Min-Max
5	Num Rotatable Bonds	Min-Max
6	TPSA	Min-Max
7	LogP	Min-Max
8	Fraction sp3 Carbons	Min-Max
9	Radius of Gyration	Min-Max
**Atom Features**		
10	Atomic Number	Min-Max
11	Explicit Degree	Value / 3
12	Aromaticity	0 = No, 1 = Yes
13	Explicit Valence	Value / 4
14	Hybridization	sp = 0, sp2 = 0.5, sp3 = 1
**Bond Features**		
15	Bond Length	Min-Max
16	Bond Type	Single = 0, Double = 1, Aromatic = 0.5
17	Conjugation State	0 = No, 1 = Yes
18	Ring Size	Value / 8

Table 1.2 Hyperparameters used in the UMPNN model for training.

**Hidden_channels**	400
**Learning_rate**	0.00033
**Weight_decay**	0.00033
**batch_size**	248
**epochs**	50
**dropout_rate**	0.25

Predictions were carried out on two virtual screening libraries, with Pan-assay interference compounds (PAINS), BRENK, and NIH filters applied to eliminate assay interference, toxic scaffolds, and chemically unstable moieties. After filtering, the Hit Locator library from Enamine and the core in-stock library from ChemBridge yielded a combined total of 1,172,240 compounds.

To ensure test-set independence, the dataset was partitioned using a Bemis–Murcko scaffold split, with entire scaffold groups assigned to the training set until approximately 80% of the data were included.

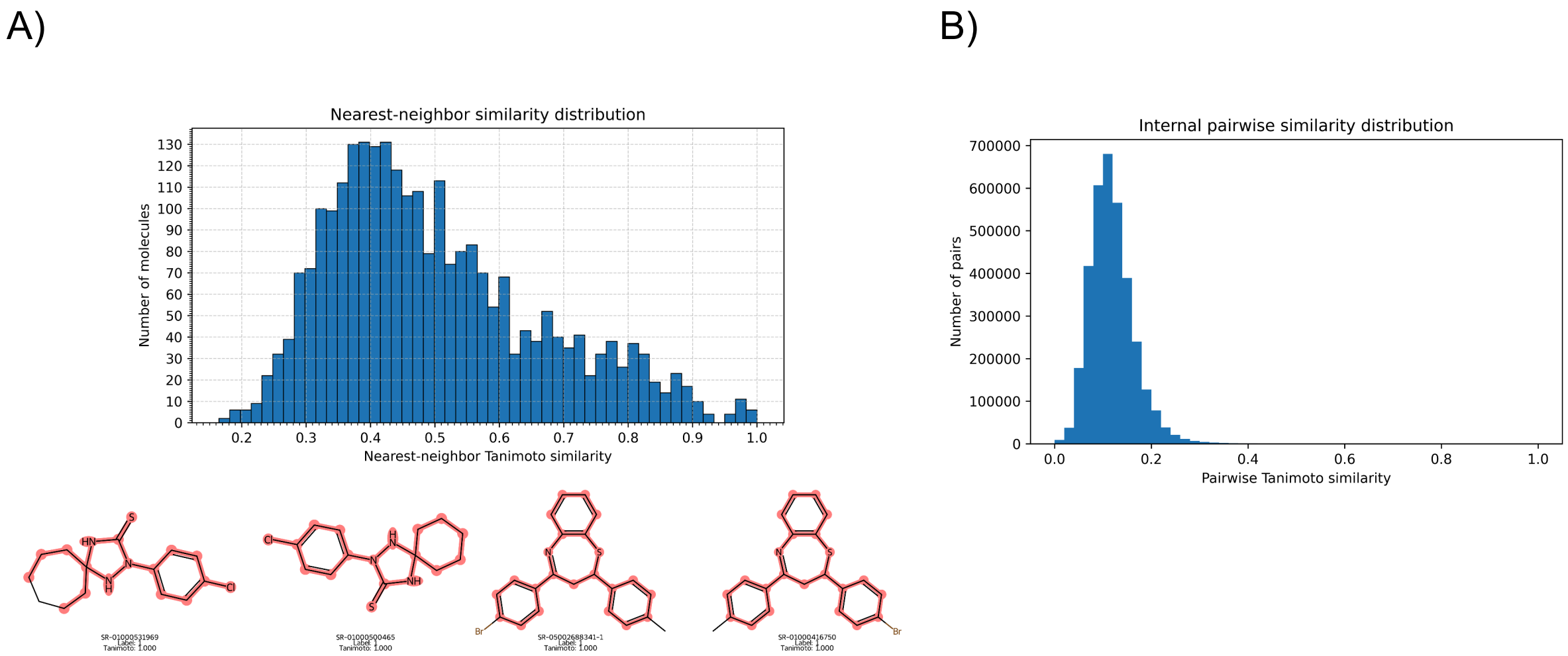

Fig. 1.3. Tanimoto similarity distributions used to assess dataset independence. A) Nearest-neighbor Tanimoto similarity distribution between test-set molecules and their closest training-set neighbors. Only two test–train pairs displayed a Tanimoto similarity of 1.0; inspection confirmed that these correspond to distinct chemical entities rather than duplicated compounds. This supports the absence of exact train/test duplication. B) Internal pairwise Tanimoto similarity distribution across the dataset, with an average similarity of approximately 0.16.

1.2. Virtual screening protocol

We performed molecular docking on the predicted inhibitors from the UMPNN using a funnel-like set of operations to narrow down the candidates for experimental testing. The target enzyme for docking was a representative conformational state (mean potential energy) derived from molecular dynamic trajectories of the nsp13 in complex with a previously identified hit: MWAC-3429[14].

This and next protein included systems underwent the same protein preparation workflow: crystallographic waters were discarded, zinc ions maintained and manually located if missing (the nsp13 natively has zinc ions), disulfide bonds were automatically created when cysteines were closed by less than 3.2 Å, bond orders were assigned, missing residues were modeled using Prime[38] to generate a continuous chain.
Protonation states of ionizable residues were assigned according to a pH 7.4 using Epik7[36], and hydrogen-bond optimization was carried out with PROPKA[39]. The prepared structures were then energy-minimized using the OPLS4 force field to relieve local strain and optimize hydrogen orientations.

Predicted inhibitors were prepared using the LigPrep workflow in the Schrödinger suite, which included structure standardization, hydrogen addition, neutralization, ionization-state assignment, tautomer generation, stereoisomer enumeration, and 3D conformer minimization. Macro-protonation states were assigned according to the dominant macroscopic pKa population

at pH 7.4 predicted by Epik7[36], and up to 32 stereoisomers and relevant tautomeric states were generated per compound prior to docking.

We used Glide with standard precision (SP) mode to dock each molecule conformation and retained the top docking scores below - 7.5, ensuring fair binding affinity scores. The search space for molecular poses was an external box of length 15 Å and a centered inner box of 10 Å containing all the residues identified with SiteMAP[37] analysis and included residues: LYS139, GLU142-143, LYS146, ASN179-VAL181, HIS230, SER310-HIS311, ASN361, MET378 and PRO408. Subsequently, we eliminated repeated conformations for each ligand based on the Glide EMODEL score[13].

The Prime MM–GBSA (Molecular Mechanics Generalized Born Surface Area) method was employed as a re-scoring step to refine and prioritize ligand poses generated from Glide docking. We used the VSGB dielectric model working with water as a solvent. The forcefield used was the OPLS_2005. During local pose refinement, the ligand and all protein atoms within 5 Å were treated as flexible during energy minimization and a single conformation resulting from this approach was used for the calculation. Poses were retained only if the estimated binding free energy satisfied $\Delta G_{bind} < -43.7$ Kcal/mol, a cutoff informed by internal benchmarking against the previously characterized nsp13 inhibitor MWAC-3429[14].

To increase chemical diversity and thereby improve the probability of identifying a hit, compounds were clustered using a Tanimoto similarity based DBSCAN approach. Molecules were encoded as Morgan fingerprints (r=2, nbits=1024), and pairwise Tanimoto similarities were converted to distances for clustering. DBSCAN was applied with a distance cutoff of 0.5 and a minimum cluster size of two. We subsequently selected one compound per cluster and all the unclustered molecules.

To further narrow the final set of molecules for experimental testing, we evaluated substructure similarity to the reference ligands MWAC-3429, MWAC-3431, and MWAC-3439 using MCS scores. This metric provides a bounded measure of scaffold overlap and enabled the selection of 50 compounds that retained an MCS score > 0.5 with at least one of the three reference hits.

1.3. Apo and ligand-bound simulations of SARS-CoV-2 nsp13

We first simulated nsp13 under apo conditions to obtain a representative, co-factor-independent conformational ensemble for virtual screening. After identifying the most promising hits, we carried out additional simulations of each ligand bound to nsp13 in apo states to investigate binding stability and potential mechanisms of inhibition.

The simulations used the 601-residue construct of SARS-CoV-2 nsp13 solved at 1.87 Å resolution (PDB ID: 5ROB[8]), selected for its completeness and high structural resolution. Systems were constructed by docking the ligand with Glide using the same protocol described for the virtual screening.

Each structure was fully solvated with explicit TIP3P water molecules[40], followed by the addition of Na^+ and Cl^- ions to neutralize the system and reach a 150 mM ionic strength. The three structural zinc sites and their coordinating cysteine and histidine residues were parameterized with the cationic dummy atom (CaDA) approach[41].

Simulations were conducted on the Triton Shared Computing Cluster (TSCC) using Amber14SB force field[42] following this protocol: to relax the initial structures prior to molecular dynamics, a three-stage energy minimization protocol was applied. First, a strong restrained minimization was performed on all non-hydrogen solute atoms (excluding water and ions) with a harmonic restraint of 2.5 Kcal mol^{-1} $Å^{-2}$, using 18,000 total cycles (15,000 steepest descent followed by conjugate gradient). This was followed by a softer minimization in which restraints were reduced to the protein backbone heavy atoms (residues 1–601) using a total of 35,000 cycles. Finally, an unrestrained minimization of the entire system was conducted for 80,000 cycles to remove any remaining steric clashes.

Linear heating in NVT ensemble (100 to 300 K, 500,000 steps with 1 fs time-step) with backbone nitrogen, α and β carbons restrained using 2.5 Kcal mol^{-1} $Å^{-2}$ force constants. The equilibration procedure was carried out in two independent runs using NPT ensembles: a 3,000,000 steps simulation (1 fs time-step) was performed using a force constant of 0.1 Kcal mol^{-1} $Å^{-2}$ on the same atoms listed above; followed by a 40,000,000 step simulation (1 fs time-step) free of restraints was carried out ensuring a stable RMSD (no transitions above 2 Å) in the last half of the simulation. Finally the production simulation included three independent replicas in NPT ensemble (approximately 50 million steps for apo, 150 million steps for the complexes and both using 2 fs time-step) and were used for further simulation analysis.

1.4. Non-bonding interactions analysis

Representative binding poses were obtained through clustering. Hierarchical clustering was performed using the HierAgglo algorithm implemented in cpptraj[43] to identify representative conformational states of the ligand–protein complex across multiple independent production trajectories.

Prior to clustering, each frame was processed using an autoimage procedure, followed by mass-weighted centering of residues 10–591 at the origin and re-imaging under periodic boundary conditions to ensure a consistent molecular representation. Structural alignment was then carried out by computing the root-mean-square deviation (RMSD) of protein backbone $C_α$ atoms (residues 5–595) relative to an energy-minimized reference structure.

Clustering was performed using an agglomerative hierarchical algorithm with average linkage, based on the RMSD of the ligand coordinates. An ε cutoff of 0.8 Å was applied to define cluster separation. Representative ligand conformations from the most populated clusters were extracted and saved as individual PDB files for subsequent analysis.

To assess the non-bonding interaction for the leads simulations we obtained ligand contacts within a 5.5 Å perimeter and quantified occupancies for each residue throughout the trajectories, and are reported as percentages in the results section.

Hydrogen bonds were identified on a geometrical basis using the Hydrogen Bonds plugin implemented in VMD[44]. Hydrogen bonds were identified between the protein and the ligand by selecting the protein as the donor or acceptor group and the ligand as the interaction partner. Hydrogen bonds were defined using a donor–acceptor distance cutoff of 3.3 Å and a donor–hydrogen–acceptor angle cutoff of 20°. Only polar atoms (N, O, S, F) were considered in the hydrogen bond detection. Both donor–acceptor pairs and individual hydrogen bond occurrences were recorded.

Non-bonded interaction free energies for the lead compounds were estimated using Molecular Mechanics/Generalized Born Surface Area (MM/GBSA) calculations implemented in the Amber22[45]. The GB implicit solvent model (igb=5) with mbondi2 radii was employed, while all remaining parameters were kept at their default values, including an internal dielectric constant of 1.0 and an external dielectric constant of 78.5. Frames were sampled every 10 frames across the three replicas. The total change in free energy for the complex formation included van der Waals, electrostatic, polar and non-polar solvation terms and was decomposed on a residue basis for further analysis.

1.5. NanoLuciferase reporter assay

A549-hACE2 cells (BEI Resources, NR-53821) were cultured in DMEM supplemented with 10% FBS and maintained at 37 °C in a humidified incubator with 5% CO_2. Cells were seeded at 12,000 cells/well in white-bottom 96-well plates in 45 µL of complete media and incubated overnight. The following day, 30 µL of test compounds diluted in drugging media (DMEM + 10% FBS, Pen/Strep, and 15 mM HEPES) were added. After a 2-hour pre-incubation, 15 µL of SARS-CoV-2-nLuc virus (USA-WA1/2020strain expressing NanoLuc; BEI NR-54003) diluted in virus infection media (DMEM + 10% FBS + 15 mM HEPES) was added to each well at an MOI of 0.05 (600 pfu/well), except for column 1 which received virus-free media as a negative control. NanoLuc is a luminescent reporter enzyme (19.1 kDa) used to quantify viral replication. Viral infection leads to increased luminescence, whereas antiviral compounds reduce replication and thereby lower the signal. After 48 hours post-infection, 90 µ L of Nano-Glo(TM) Luciferase Assay reagent (Promega N1110) was added to each well and incubated for 5 minutes at room temperature with gentle mixing. Luminescence was measured using a Cytation 1 reader. Percent inhibition was calculated relative to signal from virus-only (100% infection, column 12) and no-virus (0% infection, column 1) controls. EC values were determined as the concentration of compound required to reduce luminescence by 50%, reflecting inhibition of viral replication.

1.6. Cytotoxicity and viability assays in A549-hACE2 and Vero76 cells

A549-hACE2 cells (BEI-NR-53821) were cultured in DMEM supplemented with 10% fetal bovine serum (FBS) and 10 µg/mL blasticidin, maintained at 37 °C in a humidified incubator with 5% CO2. Cells were passaged twice weekly at a 1:5 to 1:10 split ratio. For viability assays, cells were plated onto white-bottom 96-well plates at a density of 12,000 cells in 45 µL/well and incubated overnight. The following day, 30 µL of test compound, diluted in drugging media (DMEM + 10% FBS, penicillin/streptomycin, and 15 mM HEPES, was added to each well. Approximately two hours later, 15 µL of additional media was added. Wells F12, G12 and H12 received 0.01% Triton X-100 as a cytotoxicity control.
After three days of incubation, 90 µL of CellTiter-Glo (Promega) reagent was added per well, incubated for 5 minutes with gentle mixing, and luminescence was recorded using a Cytation 1 instrument to assess cell viability relative to untreated controls. Vero76 cells (ATCC CRL-1587) were cultured in MEM-E medium supplemented with 10% FBS. Cells were plated similarly at 12,000 cells in 45 µL/well on white-bottom 96-well plates and incubated overnight. The next day, 30 µL of test compound, diluted in drugging media (MEM-E + 10% FBS, penicillin/streptomycin, and 15 mM HEPES), was added. Approximately two hours later, virus infection media (MEM-E supplemented with 10% FBS and 15 mM HEPES) was added to the wells. After three days of incubation, CellTiter-Glo (90 µL) was added per well, incubated for 5 minutes with gentle mixing, and luminescence was measured using Cytation 1 to determine cell viability.

1.7. nsp13 dsRNA unwinding assay

Purified SARS CoV-2 nsp13 helicase Production Both His-tagged (His-nsp13) and tag-cleaved forms of SARS-CoV-2 nsp13 were produced to obtain full-length helicase. The nsp13 gene was synthesized by GenScript based on the SARS-CoV-2 Wuhan-Hu-1 isolate (GenBank accession: NC_045512) and initially cloned into a plasmid by gene synthesis. The full-length nsp13 construct (1–601 a.a) was subsequently sub-cloned into an in-house pET-based expression vector containing N-terminal expression and purification tags. The final expressed protein contains an appended N-terminal sequence (MPSDKPHHHHHHHHHHLLENLYFQG), in which the underlined segment corresponds to the TEV protease recognition site.

Recombinant protein was expressed in E. coli BL21(DE3) at 18 °C overnight and purified using standard His-tag affinity chromatography (Qiagen Ni-NTA, 10 mL custom-packed column), followed by cation-exchange chromatography (GE Healthcare SP, 5 mL) and size exclusion chromatography (Superdex 200, 26 × 60). The purification buffer consisted of 100 mM NaCl, 10 mM HEPES (pH 7.4), and 1 mM DTT.

For preparation of tag-cleaved nsp13, the His-tagged protein was digested with TEV protease, and the reaction mixture was subjected to additional size-exclusion chromatography to remove the cleaved tag and residual TEV protease. The final purified protein was exchanged into a buffer containing 100 mM NaCl, 10 mM HEPES (pH 7.4), and 1 mM DTT.

6µL of SARS-CoV-2 nsp13 diluted for a final concentration of 500 pM in RNA helicase reaction buffer (20 mM HEPES pH 7.4, 7.5 mM $MgCl_2$, 100 mM NaCl, 2 mM ATP, and 0.05% (%v/v) BSA) was plated into each well of a black, skirted 96-well plate on ice. 2 µL of the serially diluted test compounds (final concentration range: 0.391–50 µM) were added to the plate and let shake at 300 rpm for 10 mins at 4°C on a MultiTherm Shaker (Benchmark Scientific) to allow compound–protein interactions to occur, if any. Negative-control wells received 2 µL of stop solution (20 mM HEPES pH 7.4, 0.2 M NaCl, 0.2 M EDTA) instead of compound.

The FAM/IBQ-labeled dsRNA (**Table 1.3**) was made by mixing equimolar FAM-labeled and IBQ-labeled RNA strands (IDT) in RNA Annealing Buffer (10 mM Tris HCl (pH 8.0) and 10 mM KCl) and annealing for 5 minutes at 80°C, snap cooling on ice for 1 min, and incubating for an hour at room temperature. 6µL of the dsRNA substrate diluted for a final concentration of 50nM in the RNA helicase reaction buffer was added to each well, and the plate was incubated at the same conditions for another 10 minutes. Finally, 6 µL of the Trap RNA (IDT) diluted for a final concentration of 500 nM in the RNA helicase reaction buffer was added to each well, and the plate was incubated at the same conditions for a final 10 minutes.

The plate was read on a Cytation 1 (BioTek) for 1 hour at 30°C with kinetic readings taken every 2 minutes using Ex/Em = 485/528 nm. The percent activity was determined by normalizing the data with the 100% activity control (no compound) and the 0% activity control (stop solution) at the 30 min time-point.

Table 1.3. Sequences of fluorescently labeled RNA oligonucleotides used in this study.

Oligonucleotide	Type / Labeling	Sequence (5′–3′)
FAM-U0D18	RNA, 5′-FAM	56-FAM/rArCrCrArCrGrArGrCrUrUrGrUrCrArCrUrG
U18D18-IBQ	RNA, 3′-IBQ	rUrUrUrUrUrUrUrUrUrUrUrUrUrUrUrUrUrUrArUrUrCrArGrUrGrArCr ArArGrCrUrCrGrUrGrGrU-IBQ
Trap RNA	RNA trap strand	rCrArGrUrGrArCrArArGrCrUrCrGrUrGrGrU

1.8. SARS-CoV-2 cytopathic assays

A549-hACE2 (BEI, NR-53821) were maintained in DMEM with 10% FBS in an incubator with 5% CO2 at 37 °C. Cells were passaged twice a week at a split ratio of 1:5 1:10. Cells were plated onto white bottom 96-well cell culture plates at a density of 12,000 cells/45 µL/well, and incubated overnight. The next day, 30 µL of test compound diluted in a drugging media (DMEM supplemented with 10% FBS, Pen/Strep, and 15 mM of HEPES) were added to the cell plates. Approximately two hours later, SARS-CoV2 (strain, USA-WA1/2020, BEI NR-52281) SARS-Related Coronavirus 2 Isolate) diluted in a virus infection media (VIM, DMEM supplemented with 10% FBS and 15 mM HEPES) was added to the cell plate at an MOI of 0.05 (600 pfu/15 µL/well) except the column 1, which serves as the no virus control. For column 1,

VIM was used in place of a diluted virus. After three days post infection, Cell Titer Glo (Promega) was added to each well (90 μL) and incubated for 5 min with a gentle mixing. Luminescence signal was measured with Cytation 1, and the luminescence signal and the normalized percent control cell viability (column 1 for 100% and column 12 for 0%) were recorded.

1.9. Liver Microsomal Stability (LMS)

Multi-species liver microsomal stability (LMS) assays were conducted to evaluate the susceptibility of compounds to hepatic first-pass metabolism. Assays were performed in human, mouse, and rat liver microsomes. Compounds were incubated with microsomal preparations in the presence of an NADPH regenerating system, and aliquots were sampled over time. Half-life ($t_{1/2}$) values and intrinsic clearance (CL_{int}) were determined by comparison to a marketed reference drug. In addition, metabolite identification was performed using tandem mass spectrometry (MS/MS), allowing structural insights that could guide subsequent structure–activity relationship (SAR) exploration.

1.10. Cytochrome P450 inhibition assay

CYP450 inhibition studies were conducted against the four major human isoforms: CYP3A4, CYP2D6, CYP2C9, and CYP1A2. Compounds were screened at 10 μM, and percent inhibition was determined relative to control incubations. An inhibition value of < 50% at 10 μM was set as the desired cutoff for progression. For advanced leads, CYP3A4 induction potential was evaluated by Cyprotex LLC using a pregnane X receptor (PXR) activation assay, where < 30% activation at 10 μM was considered acceptable.

Appendix A

A) MWAC-3972

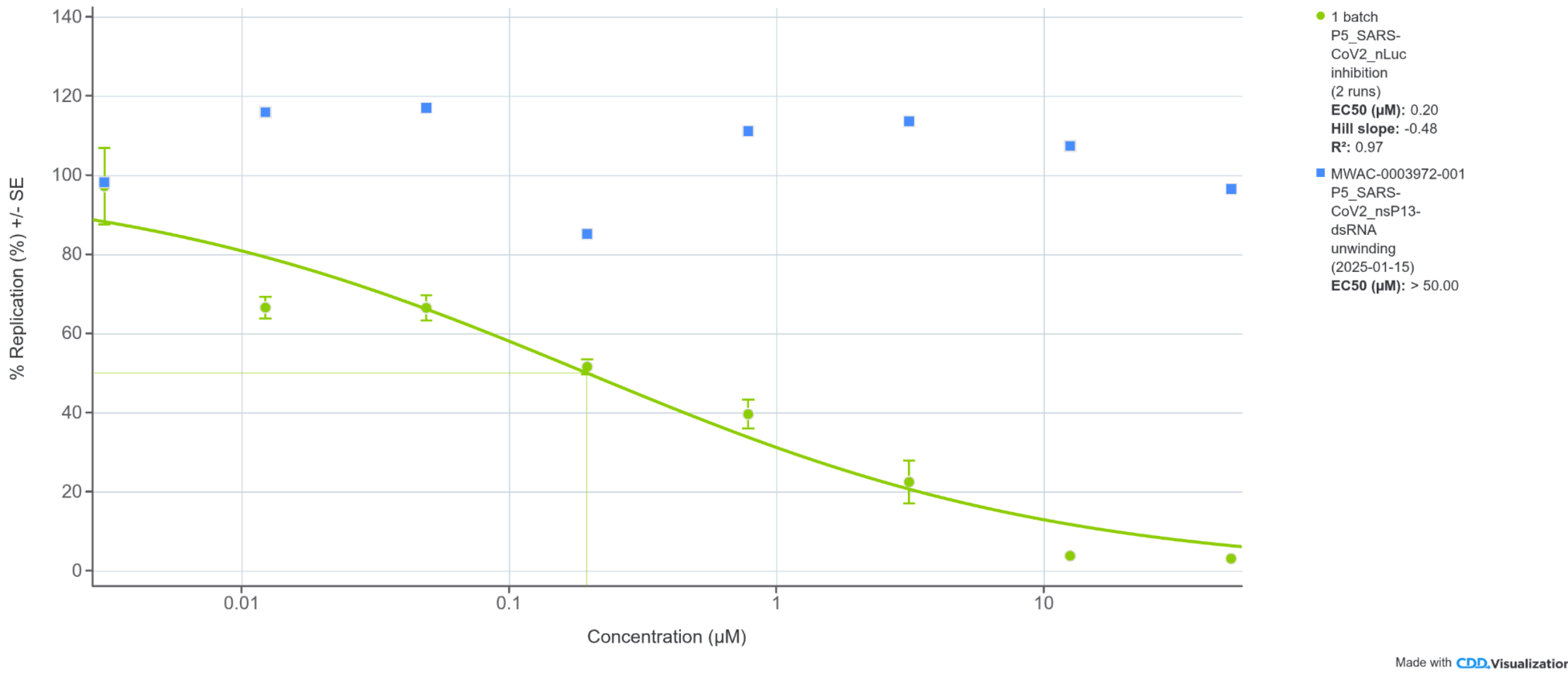

B) MWAC-3963

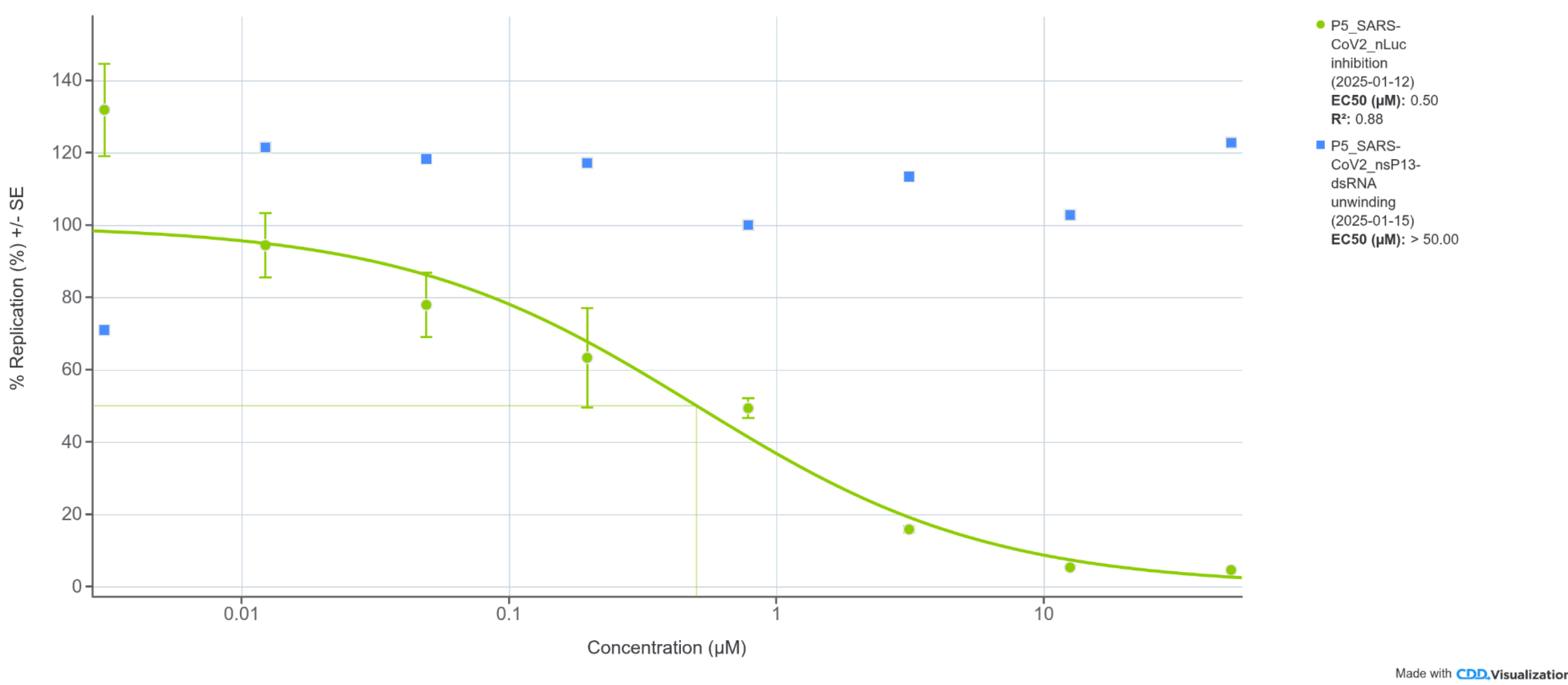

Appendix A1. Dose-response curves with n=8 concentration tested spanning 0.01-15 µM for both nLuc and dsRNA unwinding inhibition of the nsp13 for A) MWAC-3972 and B) MWAC-3963

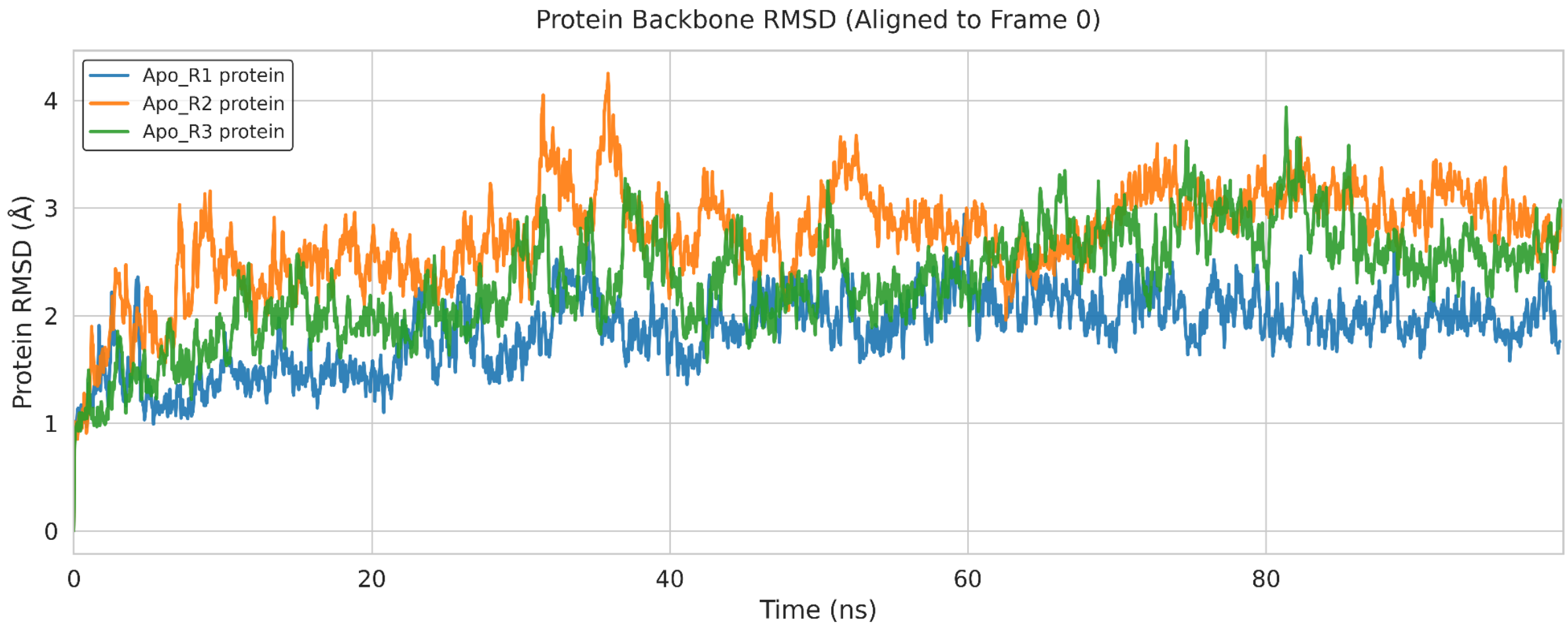

Appendix A2. Root-mean-square deviation (RMSD) of the protein backbone atoms (CA, C, O, N) aligned to the first frame after equilibration for nsp13 in the apo state. Three trajectories are shown, each representing an independent replica of approximately 100 ns of simulation time, and are colored by replica.

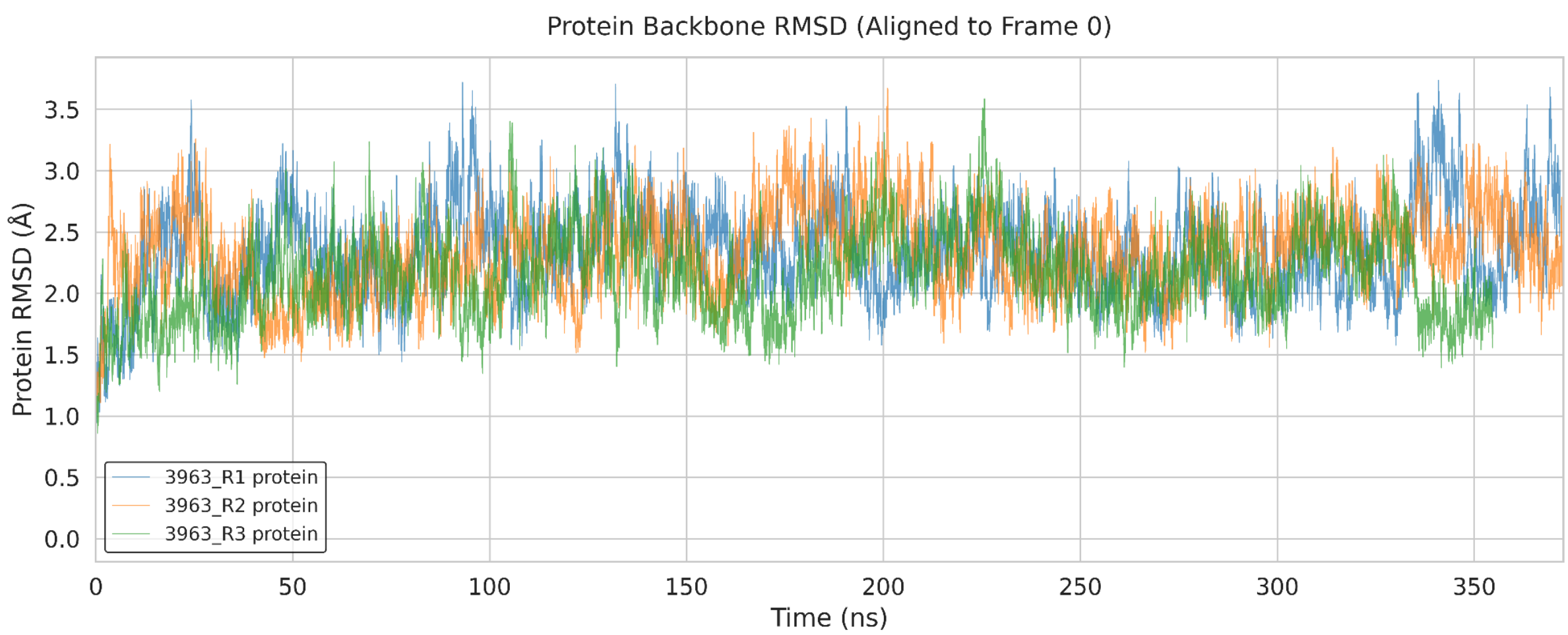

Appendix A3. RMSD of the protein backbone atoms (CA, C, O, N) aligned to the first frame after equilibration for nsp13 in complex with MWAC-3963 (charge +1). Three trajectories are shown, each representing an independent replica of approximately 375 ns of simulation time, and are colored by replica.

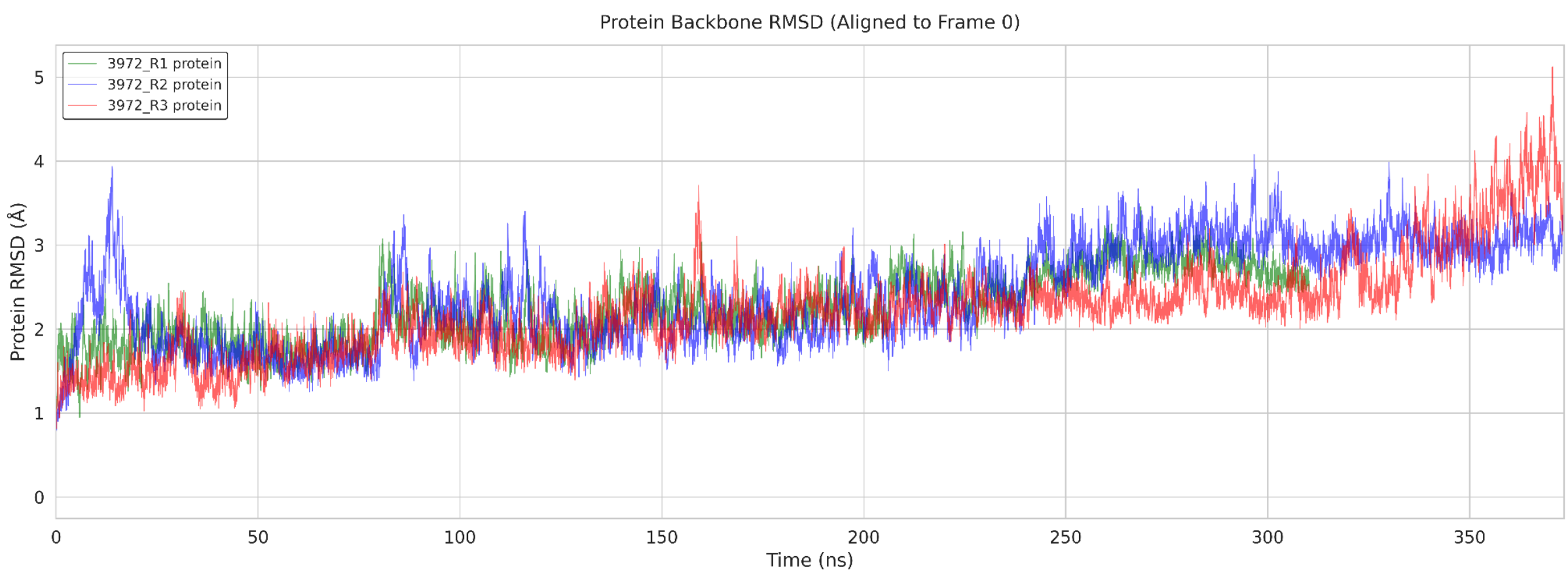

Appendix A4. RMSD of the protein backbone atoms (CA, C, O, N) aligned to the first frame after equilibration for nsp13 in complex with MWAC-3972 (charge +2). Three trajectories are shown, each representing an independent replica of approximately 375 ns of simulation time, and are colored by replica.

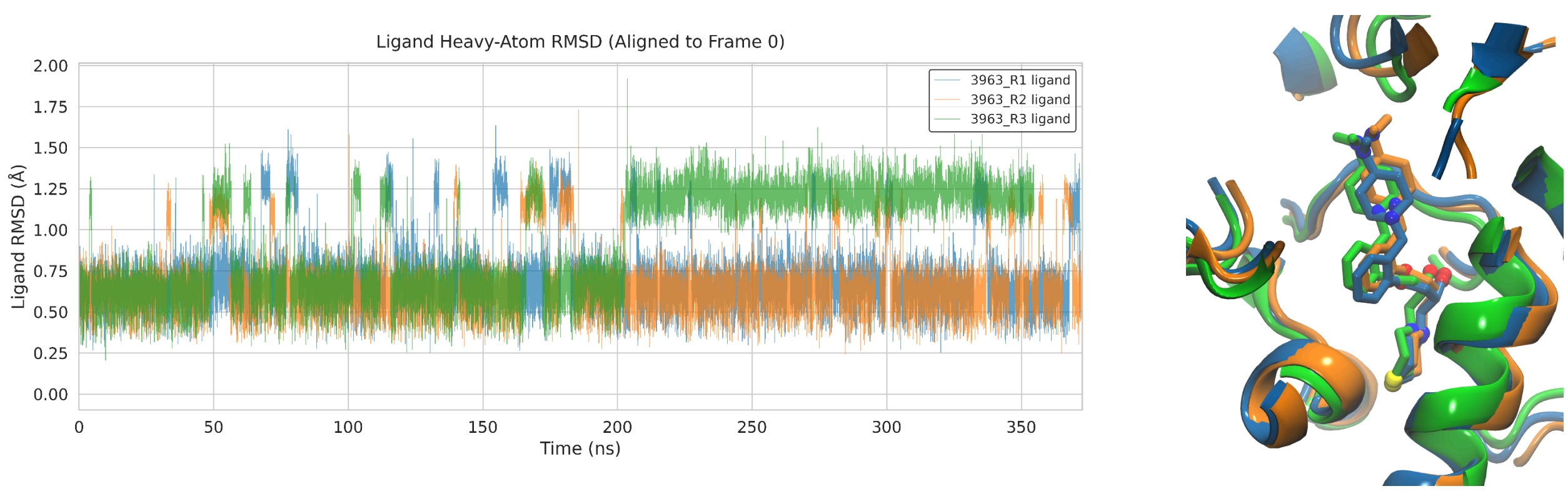

Appendix A5. RMSD of the MWAC-3963 across the three simulation replicas, computed after aligning all frames to the protein. The left panel shows the RMSD traces per replica, accompanied by a 3D representation of the ligand poses at the final frame on the right panel.

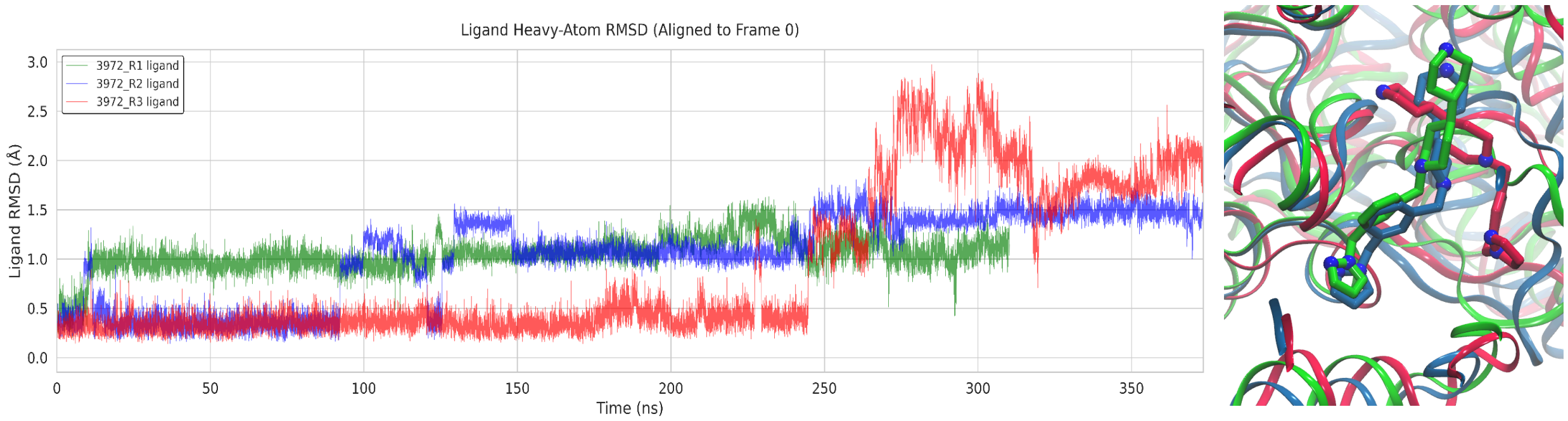

Appendix A6. RMSD of the MWAC-3972 across the three simulation replicas, computed after aligning all frames to the protein. The left panel shows the RMSD traces per replica, accompanied by a 3D representation of the ligand poses at the final frame on the right panel. In this case, the ligand remains stable throughout replicas 1 and 2, whereas replica 3 exhibits a noticeable deviation of approximately 1 Å from the stabilized replicas at the last portion of the simulation.

Appendix B. Protonation states for MWAC-3963 and MWAC-3972

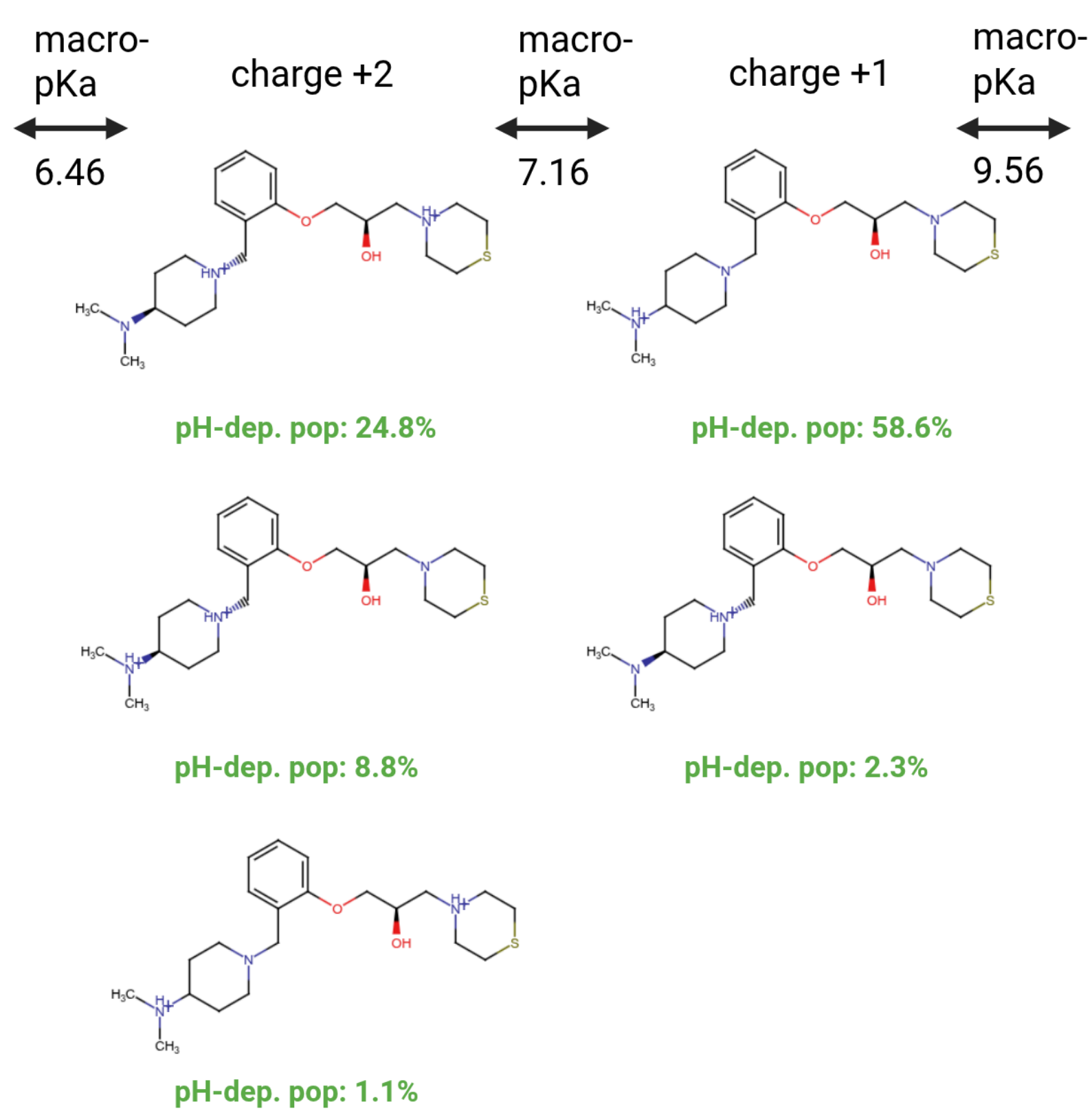

Appendix B1. Predicted protonation states and pH-dependent populations of MWAC-3963 near physiological pH using Epik v7.2 33. Macro-pKa calculations identify three titratable sites at 6.46, 7.16, and 9.56, giving rise to predominant charge +2 and +1 species. At pH 7.4, MWAC-3963 exists mainly in cationic forms (≈ 24.8 % +2 and 58.6 % +1), with minor neutral

species (< 10 %).

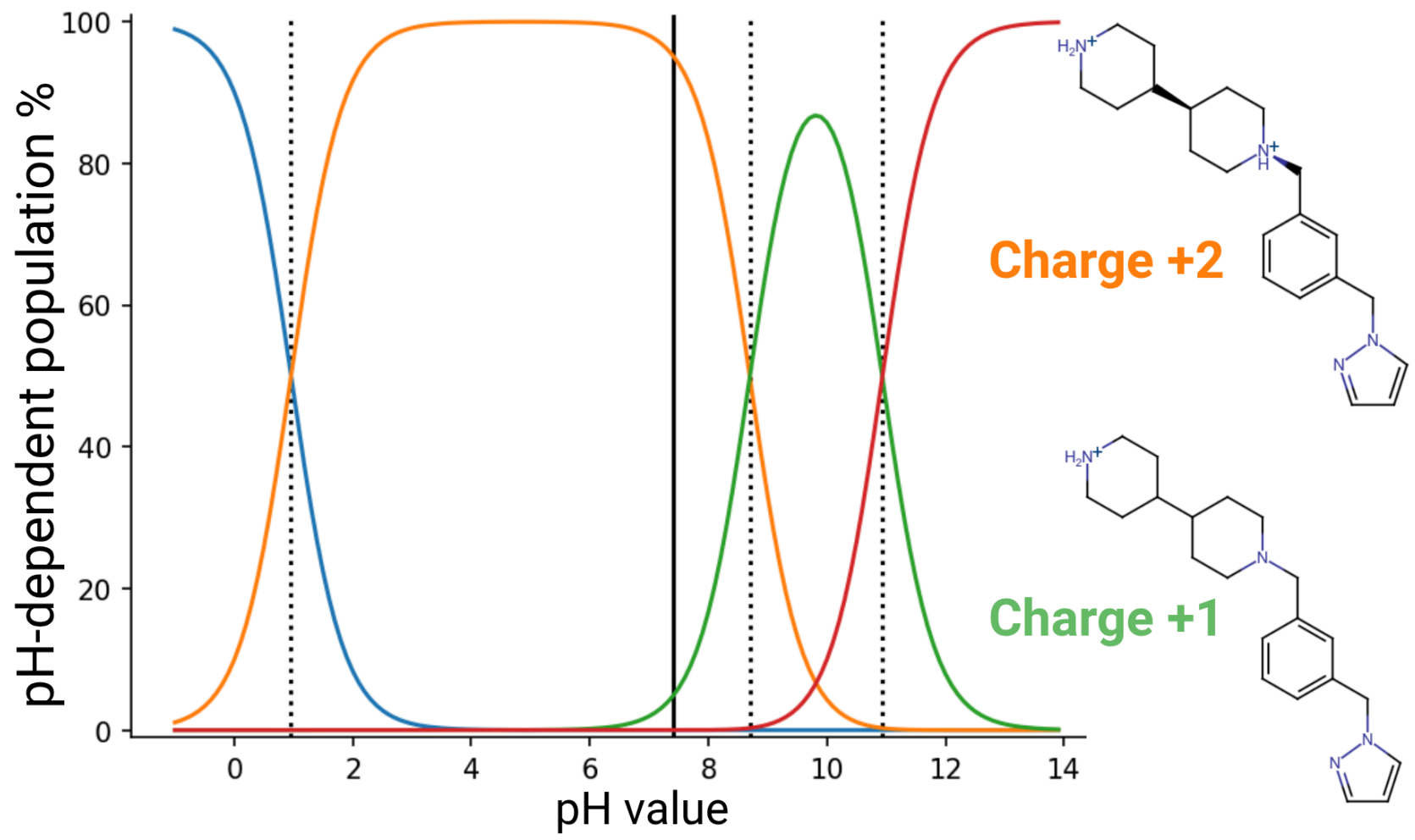

Appendix B2. Predicted pH-dependent speciation profile and predominant protonation states of MWAC-3972 using Epik v7.2 33. The speciation curves depict the relative distribution of protonation states of MWAC-3972 across the pH range. At physiological pH (7.4), the doubly protonated species (orange trace) predominates, accounting for approximately 95.2% of the total population. Three macroscopic pKa values were identified at 0.9, 8.7, and 10.9, corresponding to sequential deprotonation of the basic centers.

Appendix C. Clustering method for obtaining representatives poses

The method involves aligning all trajectory frames to a structural reference and performing hierarchical clustering based on ligand conformational similarity in *cpptraj*. First, the protein backbone (residues 5–595, Cα atoms) is aligned to the reference structure taken from the latest minimized conformation to remove overall translational and rotational motion. Following alignment, a hierarchical agglomerative clustering is applied to the ligand heavy atoms (:LIG) using a best-fit RMSD metric and a Manhattan distance criterion with an epsilon threshold of 0.8 Å. Pairwise RMSD distances between ligand conformations are computed and cached to ensure computational efficiency. Clusters are formed iteratively based on average linkage, and the representative structure for each cluster is selected as the frame with the lowest cumulative RMSD to all others in that group. The representative ligand conformations are then written to individual PDB files (DBSCAN_representative_39XX.pdb) and the largest-cluster pose is selected for non-bonding interaction analysis.

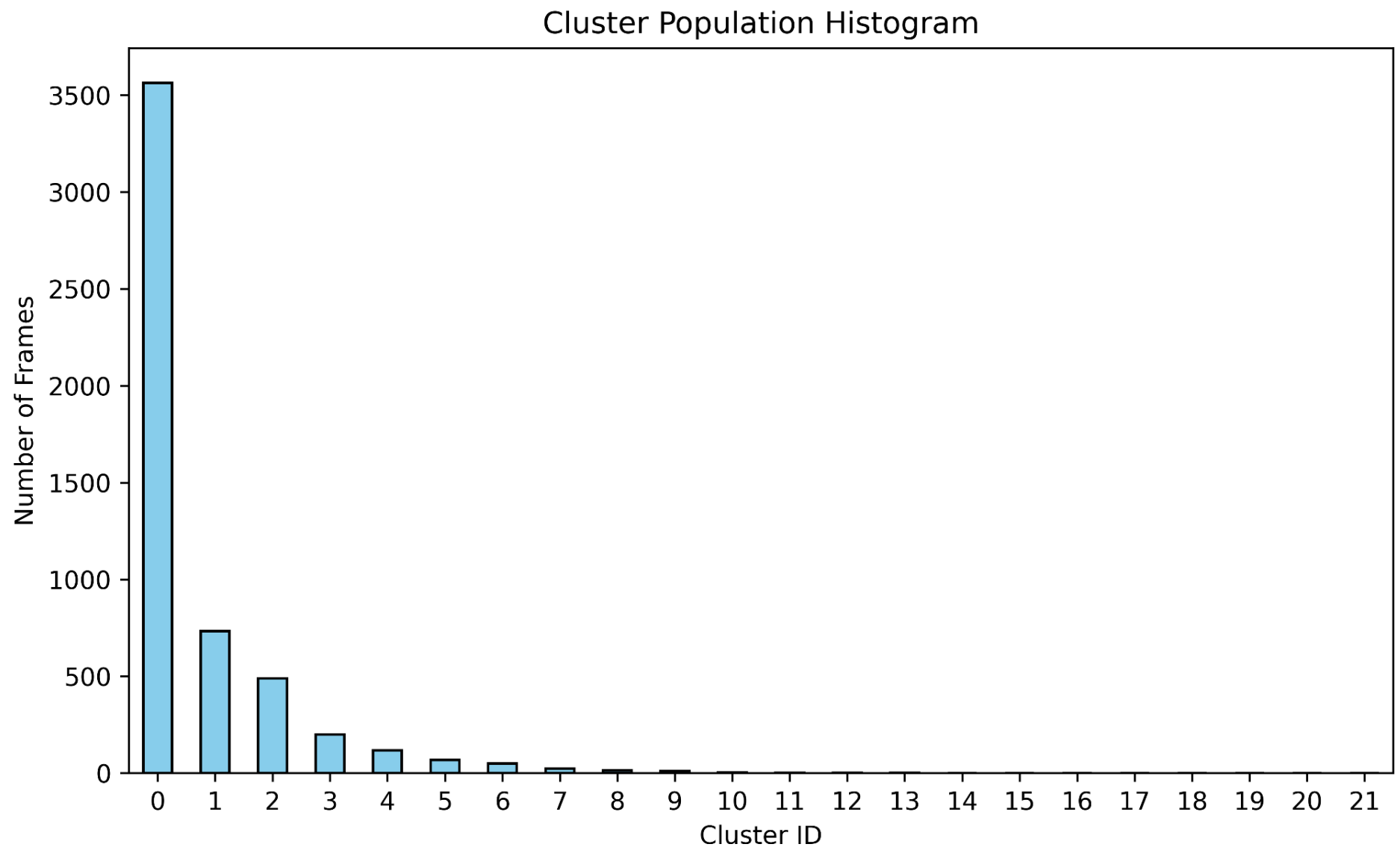

Appendix C1. Histogram of ligand poses clustered by frames for the MWAC-3972 system. These are ordered from most populated to least, where cluster 0 accounts for 67% of the simulation time and there are 9 clusters with more than 1 frame.

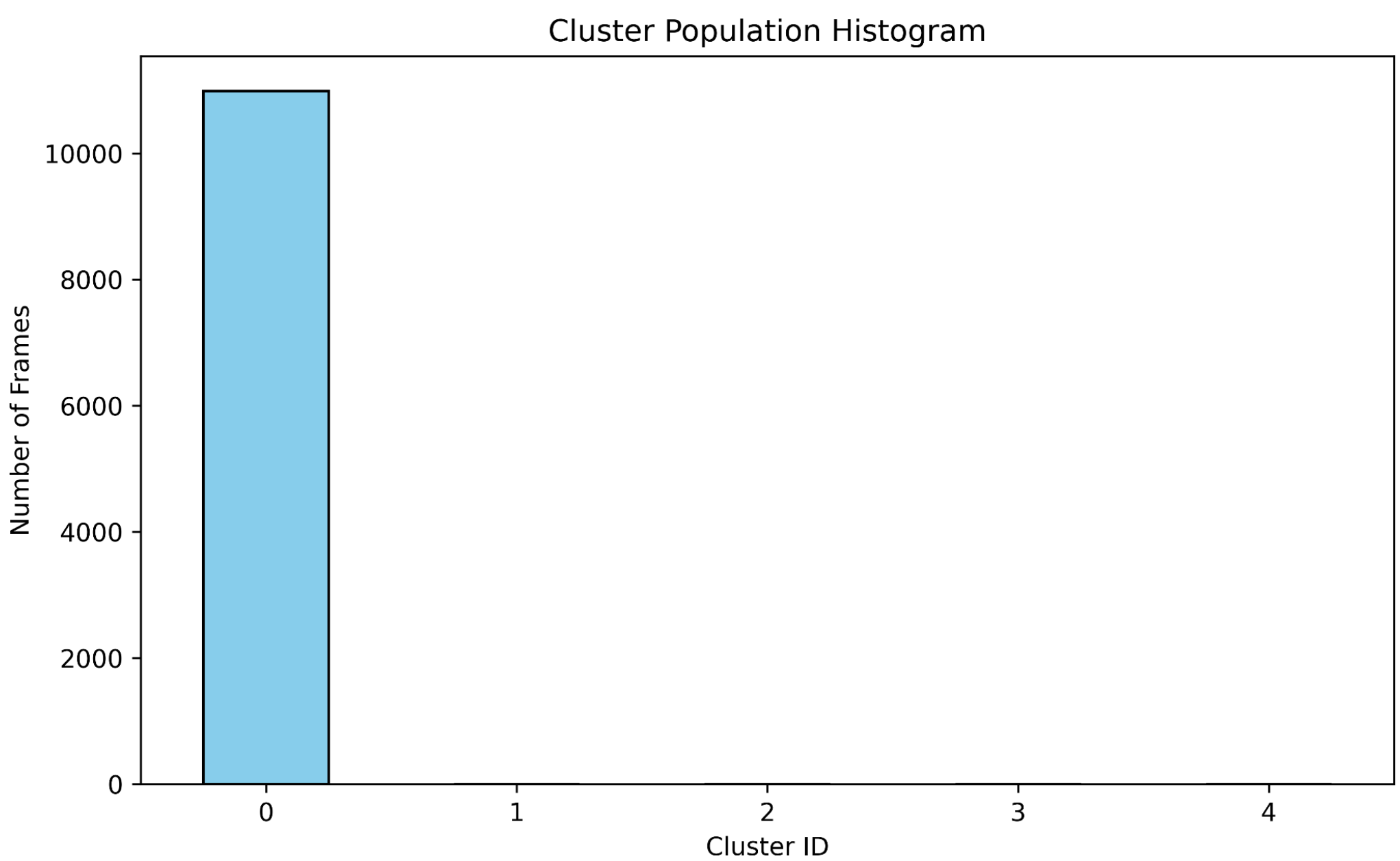

Appendix C2. Histogram of ligand poses clustered by simulation frames for the MWAC-3963 system. The three replicas exhibit highly stable binding poses, resulting in a single cluster at a permissive cutoff of ε = 0.8 in the clustering algorithm.

Appendix D. MM/GBSA and occupancy decomposition for the MWAC-3963 complex.

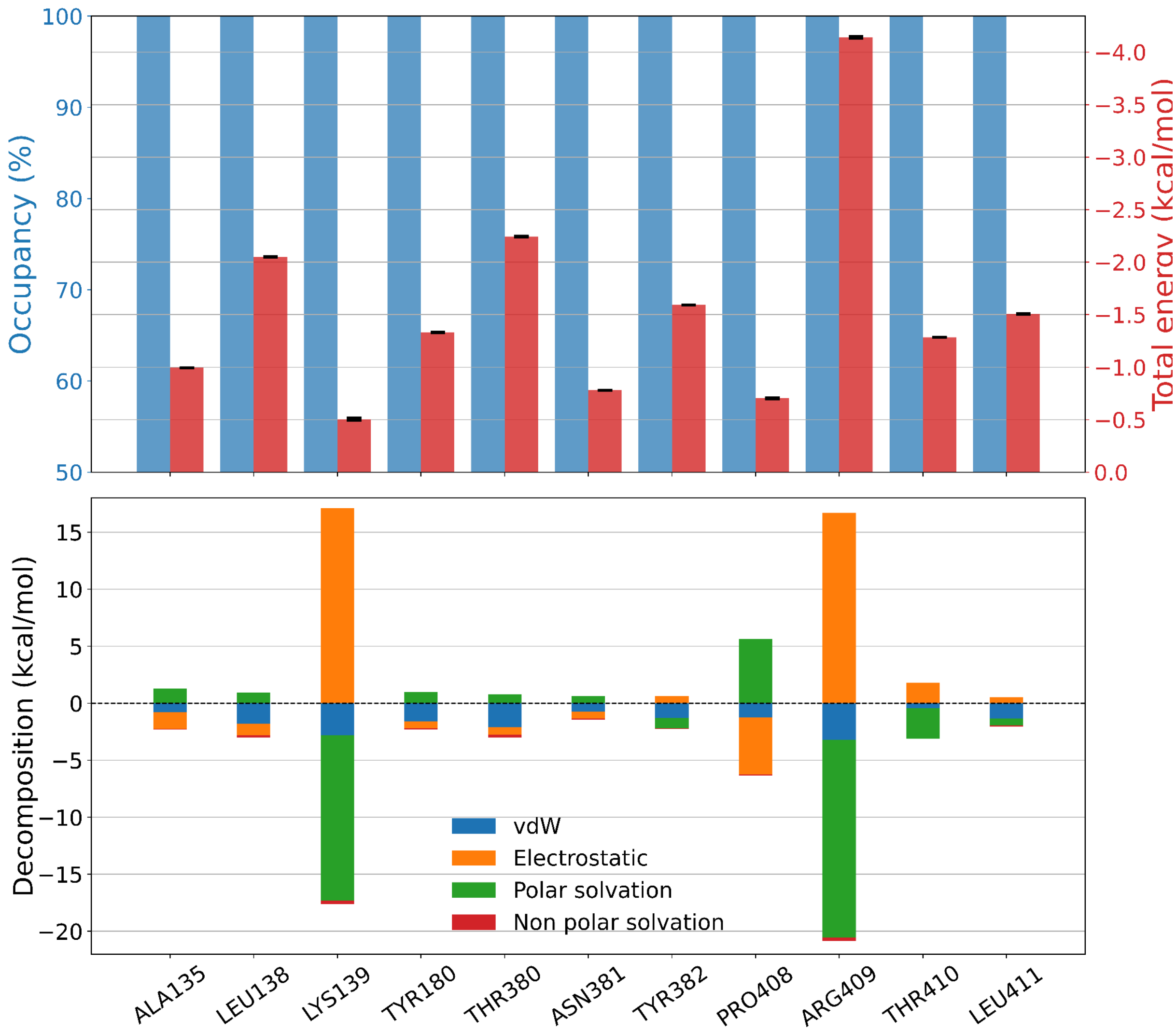

Key-interactions found for MWAC-3963 using contact occupancies, across three replicas of ≈ 350 ns each, and MM/GBSA energies. On top, Total energies in Kcal/mol (red) and occupancy across trajectories (blue) are shown side by side for the residues displaying occupancies > 50% and energies < -0.5 Kcal mol. At bottom, the decomposition in vdW (blue), electrostatic (orange), polar (green) and non-polar (red)terms are also plotted.
